# Epigenetic OCT4 regulatory network: stochastic analysis of cellular reprogramming

**DOI:** 10.1038/s41540-023-00326-0

**Published:** 2024-01-06

**Authors:** Simone Bruno, Thorsten M. Schlaeger, Domitilla Del Vecchio

**Affiliations:** 1 Department of Mechanical Engineering, Massachusetts Institute of Technology, 77 Massachusetts Avenue, Cambridge, MA 02139 USA; 2 Boston Children’s Hospital Stem Cell Program, Boston Children’s Hospital, 300 Longwood Avenue, Boston, MA 02115 USA

**Keywords:** Systems biology, Stem cells, Biochemical networks, Numerical simulations

## Abstract

Experimental studies have shown that chromatin modifiers have a critical effect on cellular reprogramming, i.e., the conversion of differentiated cells to pluripotent stem cells. Here, we develop a model of the OCT4 gene regulatory network that includes genes expressing chromatin modifiers TET1 and JMJD2, and the chromatin modification circuit on which these modifiers act. We employ this model to compare three reprogramming approaches that have been considered in the literature with respect to reprogramming efficiency and latency variability. These approaches are overexpression of OCT4 alone, overexpression of OCT4 with TET1, and overexpression of OCT4 with JMJD2. Our results show more efficient and less variable reprogramming when also JMJD2 and TET1 are overexpressed, consistent with previous experimental data. Nevertheless, TET1 overexpression can lead to more efficient reprogramming compared to JMJD2 overexpression. This is the case when the recruitment of DNA methylation by H3K9me3 is weak and the methyl-CpG-binding domain (MBD) proteins are sufficiently scarce such that they do not hamper TET1 binding to methylated DNA. The model that we developed provides a mechanistic understanding of existing experimental results and is also a tool for designing optimized reprogramming approaches that combine overexpression of cell-fate specific transcription factors (TFs) with targeted recruitment of epigenetic modifiers.

## Introduction

Through the process of cellular differentiation, embryonic stem cells evolve into a variety of specialized cell types. By contrast, cellular reprogramming converts differentiated cells to induced pluripotent stem cells (iPSCs)^1^. Since human iPSCs have functions almost identical to those of embryonic stem cells (ESCs), they can be used to replace damaged cells, representing a promising alternative to ESCs for regenerative medicine^2,3^. The first iPSC reprogramming approach, introduced by Yamanaka et al.^4,5^, is based on overexpression of four key transcription factors (TFs), OCT4, Sox2, Klf4, and c-Myc (OSKM). Because the efficiency of the initial reprogramming process was very low^6–9^, a plethora of follow-up studies have aimed at improving efficiency^10–14^.

We can largely group these studies into those that keep the TF cocktail to OSKM and investigate the extent to which the levels of these factors influence efficiency^11,15–20^ and those that add factors to the original OSKM cocktail, such as epigenetic modifiers^21–24^. Epigenetic modifiers are enzymes that either add or remove covalent modifications to histones and DNA, which ultimately affect chromatin compaction and hence gene expression. In particular, the extent to which chromatin is compactified determines how easily a gene can be transcribed^25^. The extent of chromatin compaction, in turn, is dictated by enzymatic modifications to the histones around which DNA is wrapped, such as H3K9 methylation (H3K9me3) or H3K4 methylation/acetylation (H3K4me3/ac), and by specific enzymatic modifications to DNA itself^26^. Therefore, chromatin state provides an additional layer of transcriptional regulation, which can be modulated by epigenetic modifier enzymes. Accordingly, the studies using epigenetic modifiers are grounded on the fact that in terminally differentiated cells, such as in fibroblasts used in cellular reprogramming, genes that are highly expressed in pluripotent stem cells are “shut off”, often due to highly compactified chromatin^26–30^.

In the last decade, a plethora of experimental studies investigated how chromatin modifications affect cellular reprogramming^21–24,31^. These investigations aimed to determine which chromatin modifications impact the reprogramming process in terms of improving efficiency and reducing latency variability. Here, efficiency refers to the percentage of reprogrammed cells within a given time frame and latency variability refers to variability of the time that an individual cell takes until it gives rise to a daughter iPS cell^32^. For instance, Chen et al.^22^ demonstrated that H3K9 methylation acts as a barrier during the reprogramming of mouse embryonic fibroblasts (MEFs) into iPSCs. They observed a significant increase in the number of reprogrammed cells by adding JMJD2, an enzyme that removes H3K9me3, in the OSKM cocktail. Similarly, Gao et al.^24^ showed that the inclusion of TET1, an enzyme that removes DNA methylation, to the original OSKM cocktail resulted in a twofold enhancement of iPSC reprogramming efficiency. Furthermore, it was shown that the removal of Methyl-CpG-binding domain (MBD) proteins can further increase this improvement. MBD proteins bind to methylated CpG dinucleotides^33^, protecting them from being bound by TET1^34^. In refs. ^21,31^, knock downs of MBDs were performed, resulting in more than a tenfold increase in iPSC reprogramming efficiency.

In this paper, we focus on the dynamics of reactivation of TF OCT4 during reprogramming, since it is well known that overexpression of OCT4 alone is sufficient for iPSC reprogramming^15,19,35–37^ and that OCT4 is a key regulator of TET and JMJD2^26,38–42^. To this end, we created a model for the three-gene network composed of OCT4, TET1, and JMJD2 and defined this network the *epigenetic OCT4 gene regulatory network* (Epi OCT4 GRN). We then simulated the model using Gillespie’s Stochastic Simulation Algorithm (SSA)^43^ in order to determine the efficacy of different reprogramming approaches with respect to efficiency and latency variability. We also analyzed how biochemical parameters, such as proliferation rate and the concentration of MBDs, affect the reprogramming process, with the aim of providing a mechanistic understanding of the outcomes that have been experimentally observed.

It is important to point out that a physics-based biochemical reaction model that incorporates histone modifications, DNA methylation, and transcription factor-mediated regulation has been recently developed^44^. However, this model has not yet been exploited to construct a OCT4 gene regulatory network aimed at investigating the impact of epigenetic modifiers on iPSC reprogramming. On the other side, some models that include histone modifications or DNA methylation into gene expression regulation to investigate iPSC reprogramming have appeared in the past years^45–48^. However, none of these models include both histone modifications and DNA methylation. Furthermore, none of these models include the effect of MBD proteins on TET1 activity, thus limiting the ability to study their influence on the reprogramming process.

This paper is organized as follows. In the “Results” section, we introduce the Epi OCT4 GRN and present the results of our computational analysis. Finally, in the “Discussion” section we present a discussion and conclusive remarks.

## Results

In this section, we first introduce the model of the epigenetic OCT4 gene regulatory network developed in this paper. We then provide detailed explanations of the results obtained from our computational analysis. Specifically, we first studied how DNA methylation affects the dynamics of cellular differentiation, which we captured in our model by the progressive inactivation of the OCT4 gene starting from a high OCT4 level, corresponding to the pluripotent state^49,50^. We then compared different reprogramming approaches by studying the impact of overexpressing OCT4 alone and in combination with TET1 and JMJD2 on the dynamics of OCT4 gene reactivation. Specifically, we examined process efficiency and latency variability for each approach, as these metrics have been commonly employed in published experimental studies to assess the success of cellular reprogramming^21–24,31^. To this end, we performed a computational study of the temporal trajectories of the system by simulating the reactions associated with the Epi OCT4 GRN (Fig. 2) with Gillespie’s Stochastic Simulation Algorithm (SSA)^43^.

### Model of the epigenetic OCT4 gene regulatory network

Our model includes one TF gene, OCT4, and two chromatin modifier genes, TET1 and JMJD2. We consider OCT4 as the only TF in our model because it has been shown that overexpression of OCT4 alone leads to reprogramming^15,19,35–37^. The chromatin modifiers JMJD2 and TET1 are enzymes that catalyze the erasure of histone modification H3K9me3 and DNA methylation, respectively^26,38–42^, two chromatin modifications associated with compacted chromatin state and gene silencing^51^. Specifically, while JMJD2 directly erases H3K9me3^26,38–40^, TET1 recognizes CpGme dinucleotides and converts methylated CpG to carboxylcytosine through multiple intermediate forms^41,42^, none of which is recognized by DNMT1, the enzyme responsible for copying the CpGme pattern on the nascent DNA strand during DNA replication^51^. Transcription factor OCT4 recruits writers of H3K4me3 to its own gene^52^ and to the JMJD2^53^ and TET1^54^ genes. This leads to a model in which OCT4 self-activates and also activates TET1 and JMJD2 by recruiting writers of activating chromatin modifications, while TET1 and JMJD2 self-activate and mutually activate each other and OCT4 by erasing repressive chromatin modifications. We call this system the epigenetic OCT4 gene regulatory network (Epi OCT4 GRN).

The chromatin modifiers TET1 and JMJD2 act on the chromatin modification circuit within each gene, which has been developed in ref. ^44^. This circuit includes H3K9 methylation (H3K9me3), DNA methylation (CpGme), H3K4 methylation/acetylation (H3K4me3/ac), and their known interactions. The first two modifications are associated with a repressed gene state^51^, while H3K4me3/ac is associated with an active gene state^26,55^.

**Fig. 1:**
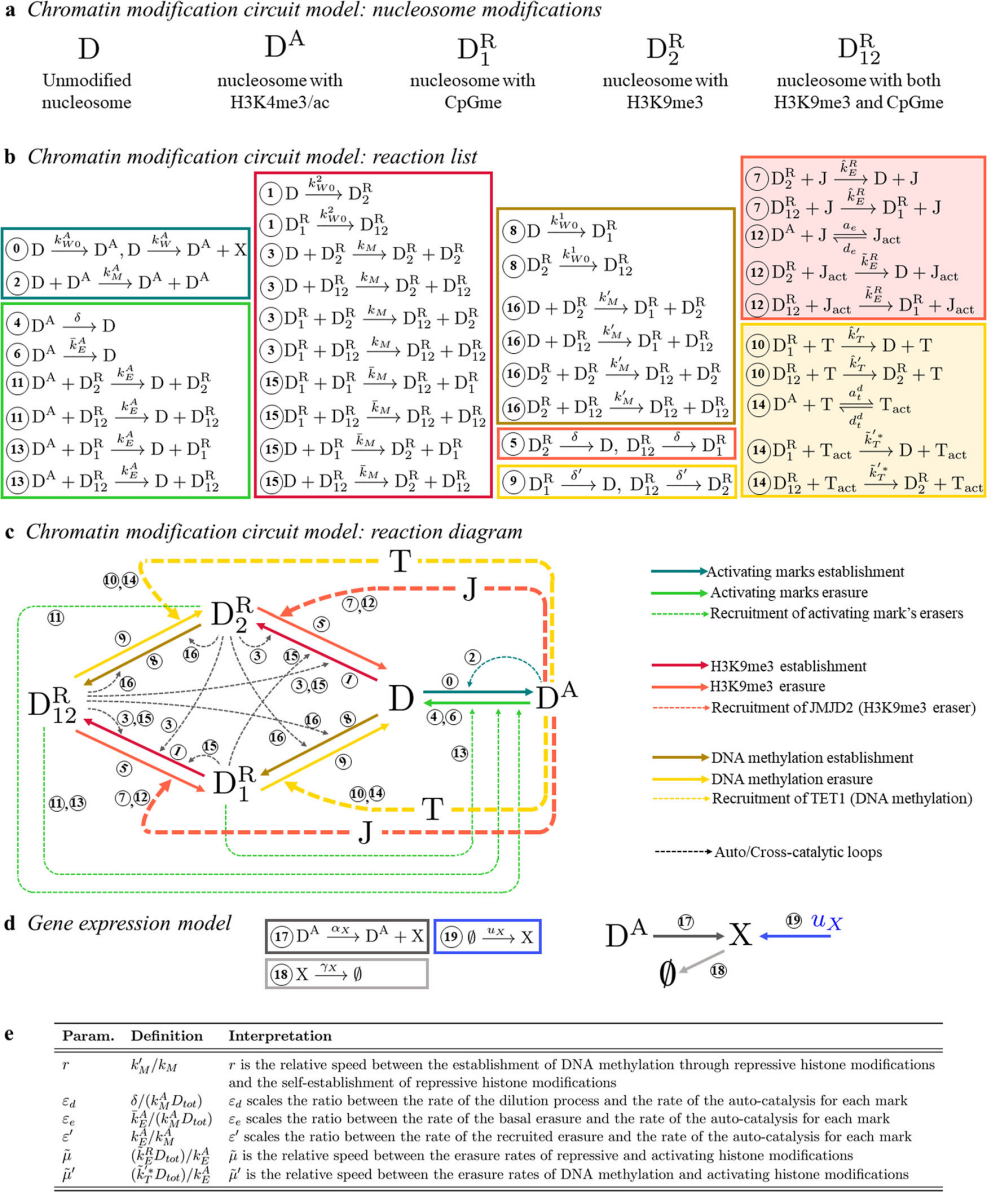
The role of TET1 (T) and JMJD2 (J) in the gene’s inner chromatin modification circuit. **a** Nucleosome modifications considered in the model. **b** Reactions associated with the chromatin modification circuit of each gene X, with X = O (OCT4), T (TET1), J (JMJD2). Here, reactions associated with activating marks, H3K9me3 and DNA methylation are enclosed in green boxes, pink boxes, and yellow boxes, respectively. Dark shades are associated with reactions describing the establishment of the modifications and light shades are associated with reactions describing the erasure of the modifications. Furthermore, shaded boxes enclose reactions involving J (pink) and T (yellow). Finally, each reaction rate constant is subscripted with *W*, *M*, or *E* to indicate the association of its corresponding reaction with the writing (establishment), maintenance (auto/cross-catalytic reactions), or erasure of a chromatin modification. **c** Diagram representing the chromatin modification circuit for each gene X. **d** Reactions associated with the gene expression model. Specifically, reactions associated with production (dark gray), dilution/degradation (light gray), and artificial overexpression *u*_*x*_ (blue) of the gene product X. The reactions are described in the “Model of the epigenetic OCT4 gene regulatory network” subsection. **e** Definitions and interpretations of *r*, *ε*_*d*_, *ε*_*e*_, ${\varepsilon }^{{\prime} }$, $\tilde{\mu }$, and ${\tilde{\mu }}^{{\prime} }$.

We next describe the chromatin modification circuit and the model of gene expression (see ref. ^44^ for details). In terms of species, in this model we have D (nucleosome with DNA wrapped around it), D^A^ (nucleosome with H3K4me3/ac), ${{{{\rm{D}}}}}_{2}^{{{{\rm{R}}}}}$ (nucleosome with H3K9me3), ${{{{\rm{D}}}}}_{1}^{{{{\rm{R}}}}}$ (nucleosome with CpGme), and ${{{{\rm{D}}}}}_{{{{\rm{12}}}}}^{{{{\rm{R}}}}}$ (nucleosome with both H3K9me3 and CpGme) (Fig. 1a). Then, the expression rate of each gene will be determined by the number of nucleosomes with activating (D^A^) or repressive (${{{{\rm{D}}}}}_{1}^{{{{\rm{R}}}}},{{{{\rm{D}}}}}_{2}^{{{{\rm{R}}}}},{{{{\rm{D}}}}}_{12}^{{{{\rm{R}}}}}$) chromatin modifications. In terms of molecular interactions, both histone modifications and DNA methylation can be de novo established (process encapsulated in reactions ⓪, ①, and ⑧). Then, histone modifications can enhance the establishment of marks of the same kind to nearby nucleosomes via a read-write mechanism, generating auto-catalytic reactions (encapsulated in ②, ③). Analogously, repressive histone modifications enhance the establishment of DNA methylation, and vice versa, generating cross-catalytic reactions (encapsulated in ⑮, ⑯). Finally, each modification can be passively removed through dilution, due to DNA replication (reactions ④, ⑤, and ⑨), or through the action of eraser enzymes (basal erasure) (reactions ⑥, ⑦, and ⑩). These erasers can be also recruited by the opposite modifications (recruited erasure), that is, repressive modifications recruit activating modification’s erasers and vice versa (reactions ⑪, ⑫, ⑬, and ⑭). In this model, the rate of the processes described above for H3K9me3 (DNA methylation) is assumed not to change if the other repressive mark is present on the same nucleosome. All the reactions described above are collected in the list of Fig. 1b, in which the reactions involving TET1 and JMJD2 are shaded in yellow and pink respectively. A diagram of the chromatin modification circuit corresponding to the reactions in Fig. 1b is provided in Fig. 1c. In this system, the transcriptional self-activation is modeled as a Hill function with cooperativity 1. Specifically, ${k}_{W}^{A}$ (Fig. 1b) is a monotonically increasing function of the abundance of X (*X*), which can be written as ${k}_{W}^{A}={\tilde{k}}_{W}^{A}(X/{K}_{A})/(1+(X/{K}_{A}))$, in which ${\tilde{k}}_{W}^{A}$ is a coefficient that does not depend on *X*, and *K*_*A*_ is the dissociation constant of the binding reaction between X and DNA^44^.

**Fig. 2:**
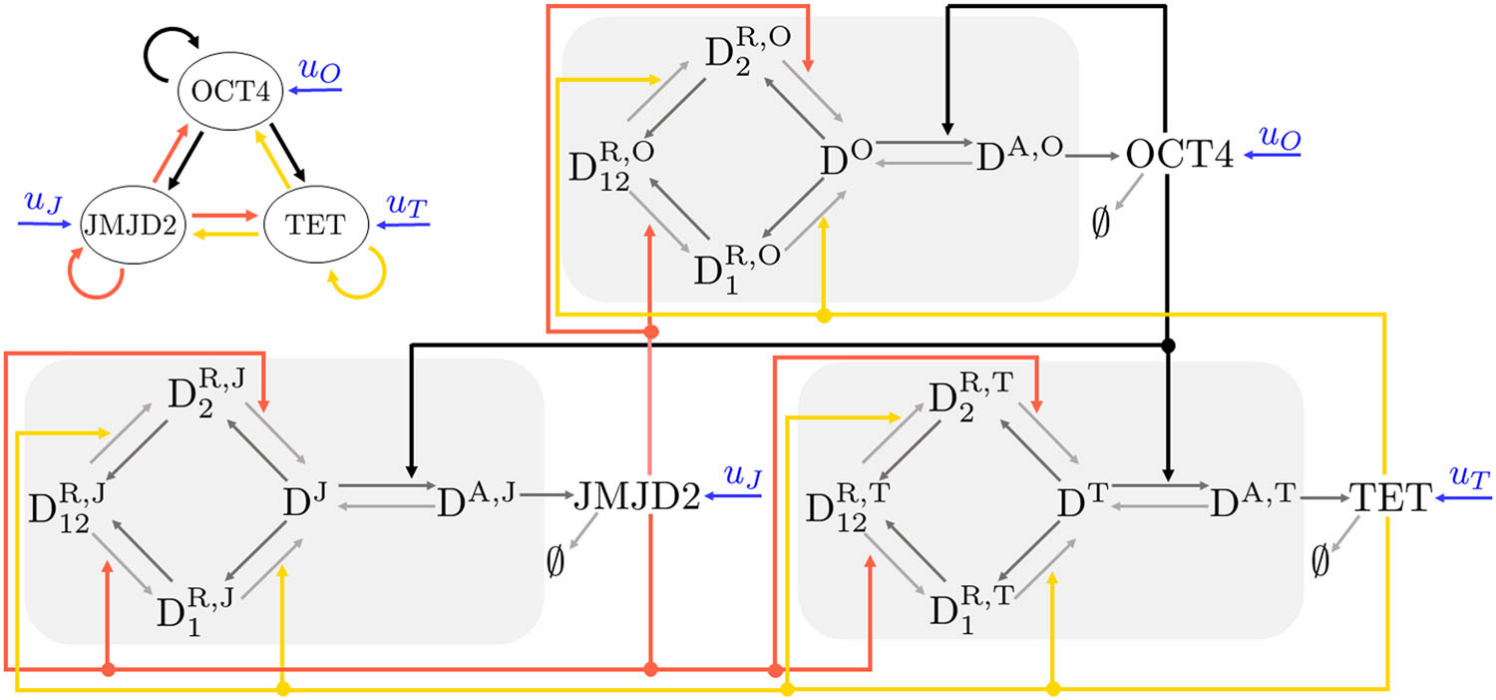
The epigenetic OCT4 gene regulatory network (Epi OCT4 GRN). Diagram of the Epi OCT4 GRN, in which OCT4 self-activates and also activates TET1 and JMJD2 by recruiting writers of activating chromatin modifications on all genes (black arrows), while TET1 and JMJD2 self-activate and mutually activate each other and OCT4 by recruiting erasers for repressive chromatin modifications on all genes (pink and yellow arrows, respectively). For simplicity of illustration, in each gene’s chromatin modification circuit we used gray for the solid arrows indicating the establishment and erasure and we did not represent the dashed arrows indicating recruitment and catalysis.

In our model, transcription is allowed only by D^A^, and transcription and translation are lumped together (reaction ⑰). The transcription by D is considered negligible because it is assumed that transcription of D by RNA polymerase II occurs concurrently with H3K4me3 deposition (i.e., conversion of D to D^A^), as observed in ref. ^56^. Furthermore, the gene product X is subject to dilution due to cell division and degradation (reaction ⑱). Finally, the production of X can be artificially increased through overexpression (reaction ⑲). A diagram representing these reactions is shown in Fig. 1d. Then, based on the interactions among OCT4, TET1, and JMJD2, we can wire the three chromatin modification circuits to obtain the Epi OCT4 GRN circuit that we analyze in this paper (Fig. 2). In the single gene’s chromatin modification circuit proposed in ref. ^44^ the concentrations of TET1 and JMJD2 impact the rate constants as they do here. However, these concentrations are constant parameters themselves. By contrast, in the Epi OCT4 GRN introduced here the concentrations of TET1 and JMJD2 are state variables and, as such, they vary dynamically under the effect of each other concentrations and the concentration of OCT4.

Let *D*_*tot*_ = D_tot_/Ω, in which D_tot_ represents the total number of modifiable nucleosomes within the gene of interest and Ω represents the reaction volume, and the normalized time $\tau =t{k}_{M}^{A}{D}_{tot}$. Let us define the dimensionless parameters $\alpha ={k}_{M}/{k}_{M}^{A}$, $\bar{\alpha }={\bar{k}}_{M}/{k}_{M}^{A}$, and ${\alpha }^{{\prime} }={k}_{M}^{{\prime} }/{k}_{M}^{A}$, in which *α* represents the normalized rate constant of the auto-catalytic reaction for repressive histone modifications, and $\bar{\alpha }$ and ${\alpha }^{{\prime} }$ represent the normalized rate constants of the cross-catalytic reactions between repressive histone modifications and DNA methylation. Let us also introduce:

1 $$r=\frac{{\alpha }^{{\prime} }}{\alpha }=\frac{{k}_{M}^{{\prime} }}{{k}_{M}},$$

that is, the ratio between the rate at which repressive histone modifications enhance the establishment of DNA methylation through cross-catalytic reactions (${k}_{M}^{{\prime} }$) and the rate at which repressive histone modifications enhance their own establishment through auto-catalytic reactions (*k*_*M*_). Furthermore, we use *η* to represent the efficiency of the maintenance process of DNA methylation by DNMT1^26^. The expression of *η* was derived in ref. ^44^ by accounting for the dilution of DNA methylation due to DNA replication and the maintenance process in which DNMT1 replicates CpG methylation on the newly synthesized DNA strand based on the pattern of the mother strand^26,57^. The expression of *η* is given by $\eta ={\delta }^{{\prime} }/\delta$, in which *δ* represents the rate constant of the passive erasure through dilution and ${\delta }^{{\prime} }$ represents the effective passive erasure rate constant obtained from the balance between the dilution and the maintenance process. In particular, *η* = 1 if DNMT1 is completely absent (no maintenance) and *η* = 0 if the maintenance process is 100% efficient. Now, we define:

2 $$\tilde{\mu }=\frac{{\tilde{k}}_{E}^{R}{D}_{tot}}{{k}_{E}^{A}},\,\,{\tilde{\mu }}^{{\prime} }=\frac{{\tilde{{k}^{{\prime} * }}}_{T}{D}_{tot}}{{k}_{E}^{A}},\,\,{\varepsilon }_{d}=\frac{\delta }{{k}_{M}^{A}{D}_{tot}},\,\,{\varepsilon }_{e}=\frac{{\bar{k}}_{E}^{A}}{{k}_{M}^{A}{D}_{tot}},\,\,{\varepsilon }^{{\prime} }=\frac{{k}_{E}^{A}}{{k}_{M}^{A}},$$

with $\tilde{\beta }=O(1)$ and $\tilde{b}=O(1)$ such that $({\hat{k}}_{T}^{{\prime} }{D}_{tot})/{\bar{k}}_{E}^{A}=\tilde{\beta }{\tilde{\mu }}^{{\prime} }$ and $({\hat{k}}_{E}^{R}{D}_{tot})/{\bar{k}}_{E}^{A}=\tilde{b}\tilde{\mu }$, respectively. Specifically, $\tilde{\mu }$ is a dimensionless parameter quantifying the asymmetry between the erasure rates of repressive and activating histone modifications, while ${\tilde{\mu }}^{{\prime} }$ is a dimensionless parameter quantifying the asymmetry between the erasure rates of DNA methylation and activating histone modifications. Furthermore, ${\varepsilon }_{d}=\delta /{k}_{M}^{A}{D}_{tot}$ is the normalized rate constant associated with dilution due to DNA replication, and, since ${\hat{k}}_{E}^{R}/{k}_{M}^{A}=\tilde{b}\tilde{\mu }{\varepsilon }_{e}$, ${\hat{k}}_{T}^{{\prime} }/{k}_{M}^{A}=\tilde{\beta }{\tilde{\mu }}^{{\prime} }{\varepsilon }_{e}$, ${\tilde{k}}_{E}^{R}{D}_{tot}/{k}_{M}^{A}=\tilde{\mu }{\varepsilon }^{{\prime} }$ and ${\tilde{k}}_{T}^{{\prime} * }{D}_{tot}/{k}_{M}^{A}={\tilde{\mu }}^{{\prime} }{\varepsilon }^{{\prime} }$, the dimensionless parameter *ε*_*e*_ (${\varepsilon }^{{\prime} }$) scales the ratio between the rate of the basal erasure (recruited erasure) and the one of the auto/cross-catalysis of chromatin modifications. We collect the definitions and interpretations of these parameters in Fig. 1e.

Finally, for modeling the gene expression process, we introduce ${\bar{p}}_{O}={\alpha }_{O}/{\gamma }_{O}$, ${\bar{p}}_{T}={\alpha }_{T}/{\gamma }_{T}$, ${\bar{p}}_{J}={\alpha }_{J}/{\gamma }_{J}$, ${\bar{u}}_{O}=({u}_{O}\Omega )/{\gamma }_{O}$, ${\bar{u}}_{T}=({u}_{T}\Omega )/{\gamma }_{T}$, ${\bar{u}}_{J}=({u}_{J}\Omega )/{\gamma }_{J}$ and ${n}_{X}^{A}$, with *X* = *O*, *T*, *J*, that is, the total amount of nucleosomes modified with activating chromatin modifications for each gene *X*.

We next describe the relationship between the abundance of MBD proteins (B) and the rate coefficients associated with the erasure of DNA methylation by the action of TET1. Specifically, these coefficients are ${\tilde{k}}_{T}^{{\prime} * }$ for the reactions in which TET1 is recruited by D^A^ (reactions ⑭) and ${\hat{k}}_{T}^{{\prime} }$ for the reactions in which TET1 is not recruited by D^A^ (reactions ⑩). As derived in^44^, ${\hat{k}}_{T}^{{\prime} }$ and ${\tilde{k}}_{T}^{{\prime} * }$ can be written as:

3 $${\hat{k}}_{T}^{{\prime} }=\frac{{F}_{1}}{{F}_{2}\frac{B}{{K}_{B}}+1},\,\,{\tilde{k}}_{T}^{{\prime} * }=\frac{{F}_{3}}{{F}_{4}\frac{B}{{K}_{B}}+1},$$

in which *K*_*B*_ is the dissociation constant of the binding reaction between B and methylated DNA and *F*_1_, *F*_2_, *F*_3_ and *F*_4_ are parameters independent of B or *K*_*B*_. Then, ${\hat{k}}_{T}^{{\prime} }$ and ${\tilde{k}}_{T}^{{\prime} * }$ increase when *B*/*K*_*B*_ decreases. From (2), ${\tilde{\mu }}^{{\prime} }$ is therefore an increasing function of ${\tilde{k}}_{T}^{{\prime} * }$ that can be written as:

4 $${\tilde{\mu }}^{{\prime} }=\frac{{\tilde{k}}_{T}^{{\prime} * }{D}_{tot}}{{k}_{E}^{A}}=\frac{{D}_{tot}}{{k}_{E}^{A}}\frac{{F}_{3}}{{F}_{4}\frac{B}{{K}_{B}}+1}=\frac{{F}_{5}}{{F}_{2}\frac{B}{{K}_{B}}+1},$$

in which ${F}_{5}={F}_{3}{D}_{tot}/{k}_{E}^{A}$ is independent of B or *K*_*B*_. From (4), we can conclude that knocking down MBD proteins (*B* = 0) or locally preventing their binding to methylated DNA (*K*_*B*_ → *∞*) allows to increase ${\tilde{\mu }}^{{\prime} }$.

### Effect of DNA methylation on the dynamics of OCT4 during differentiation

**Fig. 3:**
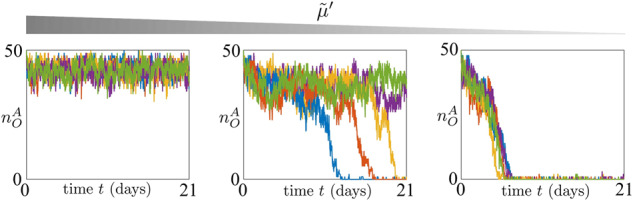
Small DNA methylation erasure rate leads to a faster and more synchronous differentiation process. Time trajectories of ${n}_{O}^{A}$ (total amount of nucleosomes modified with activating chromatin modifications for the OCT4 gene) starting from the OCT4 fully active state for different values of ${\tilde{\mu }}^{{\prime} }$. In all plots, on the *x* axis we have the time (days). The parameter values used for these simulations can be found in Supplementary Table 1. In particular, we set ${\tilde{\mu }}^{{\prime} }=1,0.15,0.07$, *ε*_*d*_ = 0.3, ${\bar{p}}_{O}={\bar{p}}_{T}={\bar{p}}_{J}=3.2$, *η* = 0.1, $\tilde{\mu }=1$, *ε*_*e*_ = 0.3 and ${\varepsilon }^{{\prime} }=1$. In our model, parameter ${\tilde{\mu }}^{{\prime} }$ quantifies the asymmetry between the erasure rates of DNA methylation and activating histone modifications. Mathematical definition of ${\tilde{\mu }}^{{\prime} }$ can be found in Eq. (2). For all simulations, we implemented the reactions listed in Fig. 1 with the SSA^43^ and we considered a time span of 21 days (*τ* = 201.6) and D_tot_ = 50 (see Supplementary Note 1).

It has been experimentally observed that, during cellular differentiation, the OCT4 gene undergoes progressive silencing, with concurrent establishment of DNA methylation^50^. Here, we investigate how the rate constant of the DNA methylation erasure process by TET1 ${\tilde{\mu }}^{{\prime} }$ affects the first time that, without any external stimulus, the OCT4 gene reaches the repressed state ${n}_{O}^{A}\approx 0$ corresponding to a differentiated state^36^, starting from the active state ${n}_{O}^{A}/{{{{\rm{D}}}}}_{{{{\rm{tot}}}}}\approx 1$ (Fig. 3). For values of ${\tilde{\mu }}^{{\prime} }$ sufficiently large, none of the trajectories reach the OCT4 repressed state in the time window of the simulation, indicating stability of the high OCT4 expression state. By reducing ${\tilde{\mu }}^{{\prime} }$, most of the trajectories reach, although in a stochastic manner, the OCT4 repressed state. Finally, if we keep reducing ${\tilde{\mu }}^{{\prime} }$, then the trajectories reach ${n}_{O}^{A}\approx 0$ quickly and in a more synchronous fashion. This result suggests that, even if the initial state, where OCT4 gene is active, is devoid of DNA methylation, the stability of this active state is contingent on a sufficiently fast erasure of DNA methylation.

Furthermore, given that TET1 is the enzyme catalyzing the erasure of DNA methylation (reactions ⑩ and ⑭ in Fig. 1b), this erasure is faster in the presence of high levels of TET1. We should then expect that stem cells have higher levels of TET1 and that, during differentiation, TET1 levels decline. This is consistent with in vivo studies showing that the level of TET1, highly expressed in embryonic stem cells (ESCs), decreases during cellular differentiation, reaching a low expression level after gastrulation^58^. This is also consistent with in vitro studies showing that in ESCs that start to differentiate, the level of OCT4 declines together with the level of TET1^59^.

Finally, it is worth noting that H3K9me3 decay rate is estimated to be much higher than the decay rate of DNA methylation^44,60^. Therefore, decreasing the erasure rate constant of repressive histone modifications $\tilde{\mu }$ has a lesser impact on both the speed of the differentiation process and the stability of the high OCT4 state compared to decreasing the erasure rate constant of DNA methylation (see Supplementary Fig. 1).

### Effect of proliferation rate on reprogramming through OCT4 overexpression

**Fig. 4:**
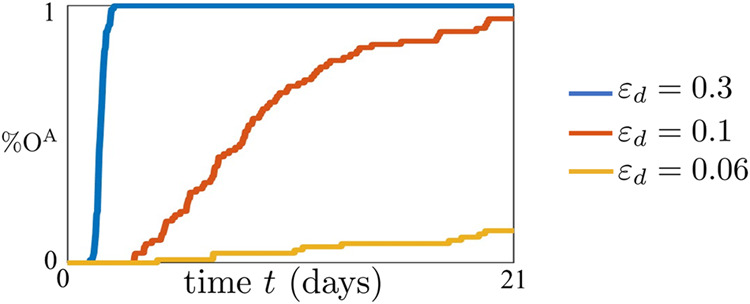
Higher proliferation rate speeds up reprogramming through overexpression of OCT4. %O^A^ for different values of *ε*_*d*_, when we set ${\bar{u}}_{0} > 0$, capturing OCT4 overexpression. The parameter values used for these simulations can be found in Supplementary Table 1. In particular, we consider *ε*_*d*_ = 0.3, 0.1, 0.06 and we set ${\bar{u}}_{0}=320$, ${\bar{u}}_{T}=0$, ${\bar{u}}_{J}=0$, ${\tilde{\mu }}^{{\prime} }=1$, $\bar{p}=5$, in which $\bar{p}={\bar{p}}_{O}={\bar{p}}_{T}={\bar{p}}_{J}$, *η* = 0.1, *ε*_*e*_ = 0.3, $\tilde{\mu }=1$ and ${\varepsilon }^{{\prime} }=1$ (see Supplementary Fig. 3 for how the parameters ${\bar{u}}_{O}$, *ε*_*e*_, ${\tilde{\mu }}^{{\prime} }$, and $\bar{p}$ influence the impact of *ε*_*d*_ on the reprogramming process). In our model, parameter *ε*_*d*_ represents the normalized rate constant associated with dilution due to DNA replication. For all simulations, we implemented the reactions listed in Fig. 1 with the SSA^43^ and we considered a time span of 21 days (*τ* = 201.6) and D_tot_ = 50 (see Supplementary Note 1).

Here, we investigate how the proliferation rate *ε*_*d*_ affects the efficiency of reprogramming when overexpressing OCT4 only. To this end, we analyzed the trajectory of the active chromatin state of the OCT4 gene, ${n}_{O}^{A}$, starting from a fully repressed state (${n}_{O}^{A}={n}_{T}^{A}={n}_{J}^{A}=0$), when we artificially overexpress OCT4, by letting ${\bar{u}}_{O} > 0$ in the reactions listed in Fig. 1d. Now, we define the process efficiency %O^A^ as the percentage of trajectories of ${n}_{O}^{A}$ that reach the active state by a prefixed time. Then, for a fixed input ${\bar{u}}_{O}$, we evaluated %O^A^ for different values of *ε*_*d*_. Simulations show that increasing *ε*_*d*_ speeds up OCT4 reactivation dynamics, making the process more efficient (Fig. 4). These findings align with the experimental results reported by Hanna et al.^32^. In their study^32^, the authors investigated the impact of various factors, including proliferation rate and addition of Nanog to the original OSKM TF cocktail, on the kinetics of iPSCs formation from somatic cells. Notably, their findings demonstrate that a higher proliferation rate accelerates the reprogramming process. The reason why increasing *ε*_*d*_ makes the OCT4 reactivation process more efficient is that a higher *ε*_*d*_ leads to a higher decay rate of all modifications (Eq. (2), Figs. 1 and 2). Given that the initial state is the OCT4 repressed state, mainly characterized by repressive chromatin marks, then higher *ε*_*d*_ leads to a faster erasure of DNA methylation and repressive histone modifications. This, in turn, allows faster establishment of activating histone modifications, leading to a faster, and also more efficient reactivation of the OCT4 gene.

### Reprogramming through concurrent OCT4 and TET1 overexpression

**Fig. 5:**
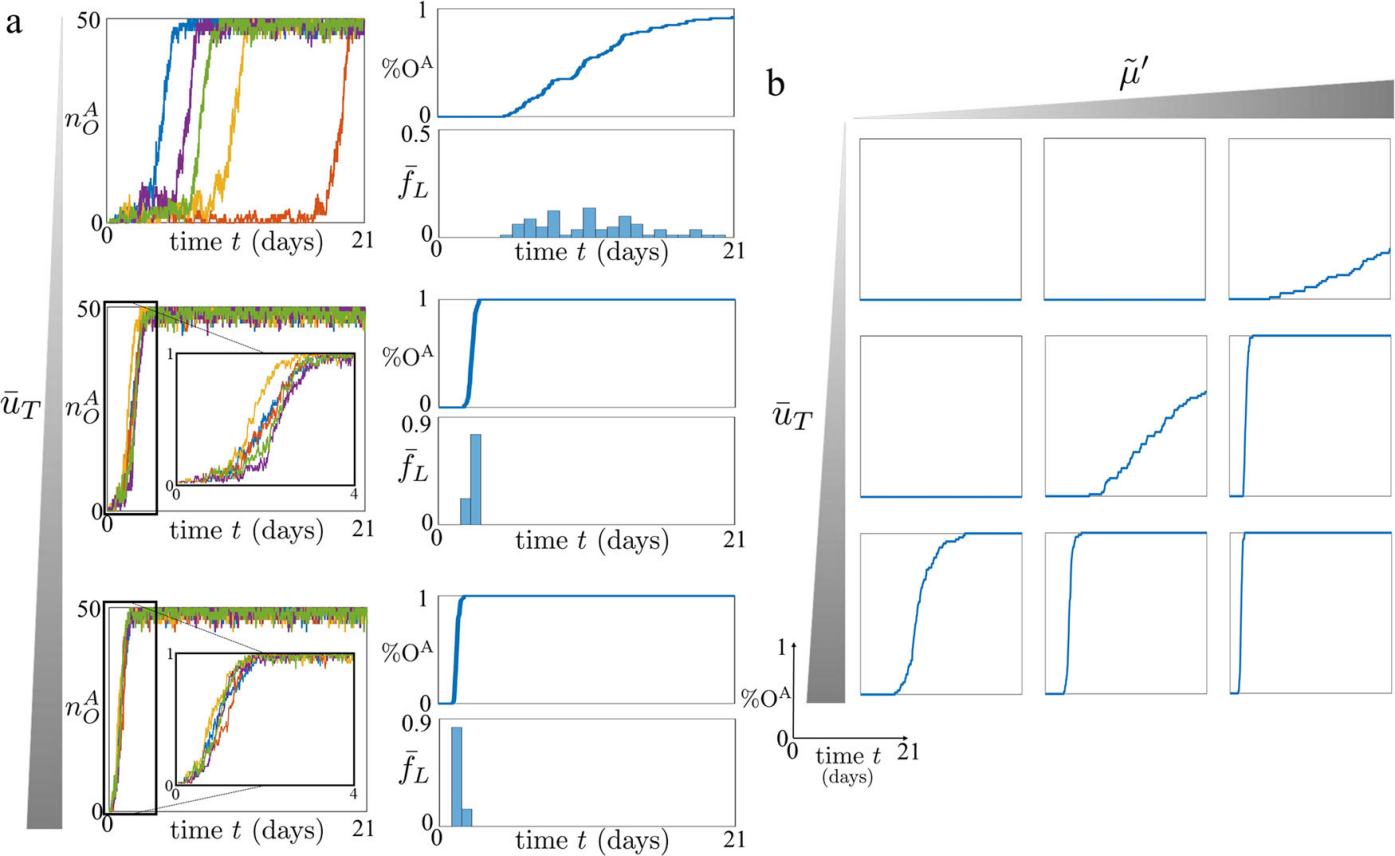
Concurrent OCT4 and TET1 overexpression leads to a more efficient and less stochastic reprogramming process, under specific parameter regimes. **a** Left hand side plots: time trajectories of ${n}_{O}^{A}$ (total amount of nucleosomes modified with activating chromatin modifications for the OCT4 gene) starting from the OCT4 fully repressed state (${n}_{O}^{A}=0$) for different values of ${\bar{u}}_{T}$. Right hand side plots: %O^A^, that is, the normalized amount of *N* = 100 time trajectories which reach ${n}_{O}^{A}\ge 40$, starting from ${n}_{O}^{A}=0$, and ${\bar{f}}_{L}$, that is, the normalized frequency of a latency value across all the *N* = 100 simulations. In all plots, on the *x* axis we have the time (days). The parameter values used for these simulations can be found in Supplementary Table 1. In particular, we consider three values of ${\bar{u}}_{T}$ (i.e., ${\bar{u}}_{T}=0,50,160$), and we set ${\bar{u}}_{O}=320$, ${\bar{u}}_{J}=0$, ${\tilde{\mu }}^{{\prime} }=0.5$, *ε*_*d*_ = 0.2, *ε*_*e*_ = 0.2, ${\bar{p}}_{O}={\bar{p}}_{T}={\bar{p}}_{J}=5$, *η* = 0.1, $\tilde{\mu }=1$ and ${\varepsilon }^{{\prime} }=1$ (see Supplementary Fig. 4 for how the parameters ${\bar{u}}_{O}$, *ε*_*d*_, *ε*_*e*_, ${\tilde{\mu }}^{{\prime} }$, and $\bar{p}$ influence the impact of ${\bar{u}}_{T}$ on the reprogramming process). **b** %O^A^ for different values of ${\tilde{\mu }}^{{\prime} }$ and ${\bar{u}}_{T}$. The parameter values used for these simulations can be found in Supplementary Table 1. In particular, we consider ${\bar{u}}_{T}=0,50,160$, ${\tilde{\mu }}^{{\prime} }=3,0.5,0.2$, *ε*_*d*_ = 0.1, *ε*_*e*_ = 0.1, and all the other parameter values equal to the ones considered for the simulations in (**a**) (see Supplementary Fig. 4 for how the parameters ${\bar{u}}_{O}$, *ε*_*d*_, *ε*_*e*_, and $\bar{p}$ influence the impact of ${\bar{u}}_{T}$ and ${\tilde{\mu }}^{{\prime} }$ on the reprogramming process). In our model, ${\tilde{\mu }}^{{\prime} }$ quantifies the asymmetry between the erasure rates of DNA methylation and activating histone modifications. For all simulations, we implemented the reactions listed in Fig. 1 with the SSA^43^ and we considered a time span of 21 days (*τ* = 201.6) and D_tot_ = 50 (see Supplementary Note 1).

Here, we consider a reprogramming approach in which the enzyme TET1 is also overexpressed. We first determined how efficiency %O^A^ and the latency variability are affected by different levels of TET1 overexpression ${\bar{u}}_{T} > 0$. To this end, we conducted several simulations for a fixed ${\bar{u}}_{O}$, capturing a fixed level of OCT4 overexpression and different values of ${\bar{u}}_{T}$ (Fig. 5). Simulations show that adding overexpression of TET1 makes the OCT4 reactivation process more efficient and reduces the latency variability (Fig. 5a). Specifically, the reduction of latency variability is captured by the narrower distribution in the values of ${\bar{f}}_{L}$, that is, the normalized frequency of a specific latency value across all the simulations. This reduction can be also captured by observing the shape of the curves %O^A^–time in Fig. 5a. As the level of TET1 overexpression increases, the synchronization of OCT4 reactivation events also increases, resulting in steeper curves. Overall, these results can be justified by the fact that activating histone modifications can be established only on unmodified nucleosomes, as shown in the chromatin modification circuit diagram (Fig. 1c), and therefore the reprogramming process cannot start until repressive modifications are erased. Accordingly, while OCT4 recruits writers for activating chromatin modifications, TET1 erases DNA methylation. OCT4 overexpression can have an effect only when unmodified nucleosomes are present, which is allowed with higher probability by the presence of TET1. These results are in agreement with experimental data showing that the addition of TET1 to the original OSKM TF cocktail increases iPSC reprogramming efficiency, promoting the formation of OCT4^+^ colonies^24^.

However, in these experiments, the efficiency of the reprogramming process was not increased pronouncedly. Specifically, the authors obtained only a twofold increase in the number of OCT4^+^ colonies observed at day 13 after doxycycline (Dox) induction^24^. This is surprising if one does not consider the role of MBD proteins, which is captured in our model by Eq. (4). Specifically, according to this model, MBD proteins bind to methylated CpG dinucleotides^33^ and then protect them from being bound by TET1^34^. This, in turn, results in the erasure rate of DNA methylation not increasing substantially when TET1 is added if MBD proteins (B) level is high. Therefore, TET1 overexpression only scarcely enhances the erasure rate of DNA methylation unless MBD proteins are either knocked down or prevented from binding DNA (see expression of ${\tilde{\mu }}^{{\prime} }$ in Eq. (4)). In order to verify how different levels of MBD affect the OCT4–TET1 overexpression reprogramming approach, we thus evaluated %O^A^ for several values of ${\bar{u}}_{T}$ and ${\tilde{\mu }}^{{\prime} }$ (Fig. 5b). The higher ${\tilde{\mu }}^{{\prime} }$ is, obtained by lowering the MBD proteins level, the more efficient and less variable the reactivation process is. These results are consistent with previous experimental data showing that knock down of MBDs increases significantly the efficiency of iPSC reprogramming and reduces its latency variability^21,31^.

Our results also show that, independent of the MBD protein level, by sufficiently increasing TET1 expression, OCT4 reactivation achieves an almost constant latency and high efficiency (Fig. 5b). Although this is in principle possible, it is also plausible that in practice the high levels of TET1 required to achieve this may be toxic to the cell or may not even be practically reachable.

### Reprogramming through concurrent OCT4 and JMJD2 overexpression

Based on experimental studies conducted in the last decade, the repressive histone modification H3K9me3 seems to be a similarly crucial barrier for the reprogramming process^22,61^. Indeed, it was shown that the addition of JMJD2 in the OSKM cocktail enhances the reprogramming efficiency, resulting in a ≈1.75-fold increase in the number of iPSC colonies obtained within the experimental time frame (8 days)^22^.

**Fig. 6:**
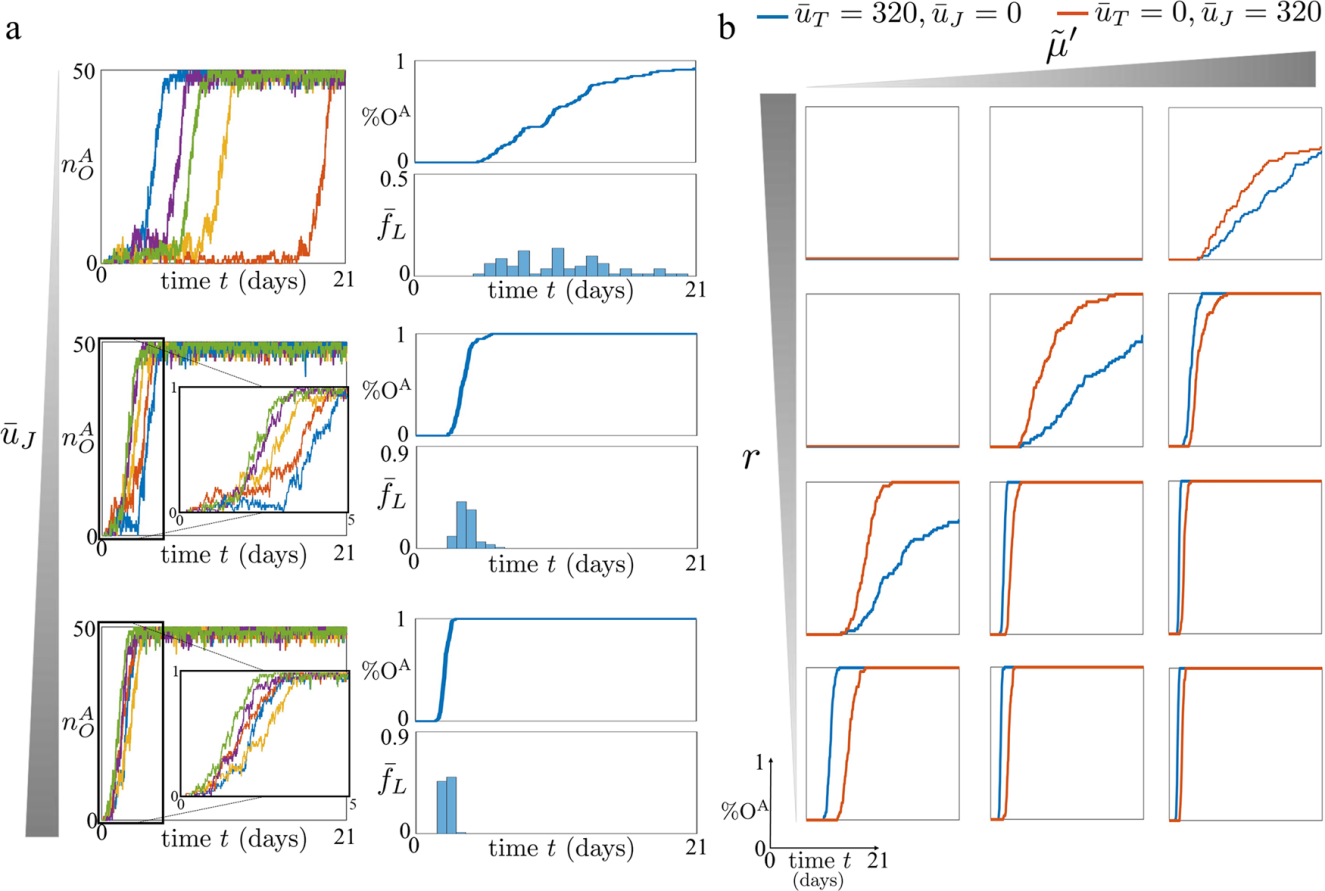
JMJD2 and TET1 overexpression can have different effects on the reprogramming efficiency and latency variability. **a** Left hand side plots: time trajectories of ${n}_{O}^{A}$ (total amount of nucleosomes modified with activating chromatin modifications for the OCT4 gene) starting from the OCT4 fully repressed state (${n}_{O}^{A}=0$) for different values of ${\bar{u}}_{J}$. Right hand side plots: %O^A^, that is, the normalized amount of *N* = 100 time trajectories which reach ${n}_{O}^{A}\ge 40$, starting from ${n}_{O}^{A}=0$, and ${\bar{f}}_{L}$, that is, the normalized frequency of a specific latency value across all the *N* = 100 simulations. In all plots, on the *x* axis we have the time (days). The parameter values used for these simulations can be found in Supplementary Table 2. In particular, we consider three values of ${\bar{u}}_{J}$ (i.e., ${\bar{u}}_{J}=0,50,160$), and we set ${\bar{u}}_{T}=0$, ${\bar{u}}_{O}=320$, ${\tilde{\mu }}^{{\prime} }=0.5$, *ε*_*d*_ = 0.2, *ε*_*e*_ = 0.2, ${\bar{p}}_{O}={\bar{p}}_{T}={\bar{p}}_{J}=5$, *η* = 0.1, $\tilde{\mu }=1$ and ${\varepsilon }^{{\prime} }=1$ (see Supplementary Fig. 5 for how the parameters ${\bar{u}}_{O}$, *ε*_*d*_, *ε*_*e*_, ${\tilde{\mu }}^{{\prime} }$, and $\bar{p}$ influence the impact of ${\bar{u}}_{J}$ on the reprogramming process). **b** %O^A^ for different values of $\tilde{\mu }$ and *r*. The parameter values used for these simulations can be found in Supplementary Table 2. In particular, we consider ${\bar{u}}_{T}=320,{\bar{u}}_{J}=0$ (blue lines), ${\bar{u}}_{T}=0,{\bar{u}}_{J}=320$ (red lines) and, for both cases ${\bar{u}}_{O}=160$, ${\tilde{\mu }}^{{\prime} }=1,0.5,0.2$, *r* = 10, 5, 1, 0.2, $\tilde{\mu }=1$, *ε*_*d*_ = 0.16, *ε*_*e*_ = 0.16, ${\bar{p}}_{O}={\bar{p}}_{J}={\bar{p}}_{T}=5$, *η* = 0.1 and ${\varepsilon }^{{\prime} }=1$ (see Supplementary Fig. 6 for how *ε*_*d*_, *ε*_*e*_, and the initial overexpression level influence the impact of ${\bar{u}}_{T}$, ${\bar{u}}_{J}$, ${\tilde{\mu }}^{{\prime} }$, and *r* on the reprogramming process). In our model, ${\tilde{\mu }}^{{\prime} }$ quantifies the asymmetry between the erasure rates of DNA methylation and activating histone modifications and *r* the ratio between the rate at which repressive histone modifications enhance the establishment of DNA methylation through cross-catalytic reactions and the rate at which repressive histone modifications enhance their own establishment through auto-catalytic reactions. Mathematical definitions of *r* and ${\tilde{\mu }}^{{\prime} }$ can be found in (1) and (2), respectively. For all simulations, we implemented the reactions listed in Fig. 1 with the SSA^43^ and we considered a time span of 21 days (*τ* = 201.6) and D_tot_ = 50 (see Supplementary Note 1).

Thus, we evaluated efficiency and latency variability for three different levels of JMJD2 overexpression, ${\bar{u}}_{J}$ (Fig. 6a). Furthermore, in order to properly compare this reprogramming approach to the one based on concurrent OCT4 and TET1 overexpression, we considered the same value as used in the previous analysis for the OCT4 overexpression level (${\bar{u}}_{O}$). Simulations show that JMJD2 overexpression reduces the latency variability of the reprogramming process and increases the number of trajectories of ${n}_{O}^{A}$ that reach the active state by a fixed time period (Fig. 6a), in agreement with the experimental data in ref. ^22^.

We next compared efficiency and latency variability of the OCT4–TET1 overexpression approach to those of the OCT4–JMJD2 overexpression approach (Fig. 6b). The results show how efficiency and latency variability of both approaches are affected by ${\tilde{\mu }}^{{\prime} }$ and $r={\alpha }^{{\prime} }/\alpha$. Parameter *r* is the ratio between the rate of the cross-catalytic reaction with which repressive histone modifications enhance the establishment of DNA methylation and the rate of the auto-catalytic reaction with which repressive histone modifications enhance their own establishment (Eq. (1)). For high values of DNA methylation erasure rate, ${\tilde{\mu }}^{{\prime} }$, the OCT4–JMJD2 overexpression approach is more efficient than the OCT4–TET1 overexpression approach only when *r* ≫ 1, that is, repressive histone modifications enhance the establishment of DNA methylation more than their own establishment (Fig. 6b). In fact, in this parameter regime, DNA methylation is rapidly erased (${\tilde{\mu }}^{{\prime} }$ large), but it is also quickly re-established by repressive histone modifications (*r* large). Thus, overexpression of JMJD2 leads to a fast erasure of repressive histone modifications. As a result, the absence of repressive histone modifications prevents the quick re-establishment of DNA methylation, leading to a rapid erasure of DNA methylation and reactivation of the OCT4 gene. In this case, using OCT4–JMJD2 overexpression for reprogramming could be more efficient and exhibit less variability in latency compared to OCT4–TET1 overexpression. For lower ${\tilde{\mu }}^{{\prime} }$, overexpression of OCT4–JMJD2 becomes more effective even with lower values of *r* (Fig. 6b). In fact, if ${\tilde{\mu }}^{{\prime} }$ is low, even a high level of TET1 overexpression may be insufficient to erase DNA methylation. However, a similar level of JMJD2 overexpression could be sufficient to erase repressive histone modifications. Then, similarly to the previous case, if the enhancement of DNA methylation by H3K9me3 is significant (*r* is sufficiently high), erasing repressive histone modifications prevents the strong re-establishment of DNA methylation by H3K9me. This leads to an acceleration in DNA methylation erasure, resulting in a faster and less stochastic reactivation of the OCT4 gene.

Overall, these results suggest that, due to the enhancement of DNA methylation by the positive reinforcement with H3K9me3, the addition of JMJD2 to OCT4 overexpression may be more effective than the addition of TET1. However, for ${\tilde{\mu }}^{{\prime} }$ sufficiently high, OCT4–TET1 overexpression approach is always more efficient than the OCT4–JMJD2 overexpression approach.

In previously conducted experimental studies, it was observed that the addition of JMJD2 to the OSKM cocktail resulted in an approximate 1.75-fold increase in the number of iPSC colonies^22^. This efficiency improvement is smaller compared to the effect achieved by adding TET1 to the OKSM cocktail, resulting in a twofold increase^24^, or even a minimum of tenfold increase when MBDs were knocked down^21,31^. These results indicate that in practical scenarios, we are likely to encounter a parameter regime where the enhancement of DNA methylation establishment through repressive histone modifications is not highly effective (low *r*). According to our theoretical findings, this leads to a situation where the OCT4–TET1 overexpression reprogramming approach is more efficient compared to the OCT4–JMJD2 reprogramming approach. Furthermore, this efficiency improves even further when MBD is reduced or knocked down (higher ${\tilde{\mu }}^{{\prime} }$).

## Discussion

In this work, we introduced the epigenetic OCT4 gene regulatory network (Epi OCT4 GRN), a network comprising a unique TF gene, OCT4, and two genes expressing chromatin modifiers, TET1 and JMJD2. The TF OCT4 and the two modifiers TET1 and JMJD2 are positively autoregulated and mutually activate each other, although through different mechanisms. Specifically, OCT4 self-activates and activates TET1 and JMJD2 by recruiting writers of activating chromatin modifications, while TET1 and JMJD2 self-activate and activate each other and OCT4 by erasing repressive chromatin modifications. Each gene also has an inner chromatin modification circuit on which the chromatin modifiers act^44^ ("Model of the epigenetic OCT4 gene regulatory network” subsection and Fig. 1c, d). Employing this model, we conducted a computational analysis to study the dynamics of reactivation of OCT4 during three reprogramming approaches based on overexpression of OCT4 alone, overexpression of OCT4 and TET1, and overexpression of OCT4 and JMJD2 by using Gillespie’s Stochastic Simulation Algorithm (SSA) (“Results” section).

Our analysis indicates that, for the same level of OCT4 overexpression, the simultaneous overexpression of either TET1 or JMJD2 increases efficiency and reduces reactivation latency variability (Figs. 5a and 6a). The correlation between higher efficiency and reduced latency variability arises from the fact that increased levels of TET1 or JMJD2 lead to a greater probability of erasing repressive chromatin marks. As a result, within a given time span, more of these erasure events take place, reducing the stochastic nature of the process. Additionally, this results in the occurrence of the same number of events within a shorter timeframe. Consequently, the overall OCT4 reactivation process becomes more efficient and exhibits decreased latency variability. Comparing these two reprogramming approaches, our results suggest that the former is more efficient if the enhancement of DNA methylation establishment by H3K9me3 is sufficiently weak (*r* sufficiently low) and DNA methylation erasure is sufficiently fast (${\tilde{\mu }}^{{\prime} }$ sufficiently large) (Fig. 6b). By comparing previously obtained experimental data, the addition of TET1 seems to be more effective than the addition of JMJD2. This observation suggests that the parameter regime characterized by a sufficiently low value of *r* and a sufficiently large value of ${\tilde{\mu }}^{{\prime} }$ is the more plausible scenario we may encounter. Physically, this parameter regime corresponds to a situation where the recruitment of DNA methylation by H3K9me3 is weak and the MBD proteins do not significantly hamper TET1 binding to methylated DNA.

We also compared the effectiveness of adding transient overexpression of TET1 or JMJD2, instead of constant overexpression (Supplementary Note 3). Our simulations show that transient overexpression of TET1 or JMJD2, although less effective than constant overexpression, still enhances the efficiency of the OCT4 reactivation process (Supplementary Figs. 7–10). Additionally, we studied the effectiveness of sequential, transient overexpression of TET1 and JMJD2. Our results show that transient overexpression of TET1 first and JMJD2 later can be as or more effective than constant overexpression of either one of the two erasure enzymes (Supplementary Figs. 11 and 12). This is because, in the parameter regime considered, the addition of JMJD2 to erase repressive histone modifications is not effective unless DNA methylation is quickly erased. To achieve a rapid erasure of DNA methylation, the addition of TET1 is necessary. More precisely, our computational analysis reveals that when the initial level of transient overexpression for both TET1 and JMJD2 is sufficiently high and matches the level of constant overexpression for either TET1 or JMJD2, the sequential transient overexpression of TET1 followed by JMJD2 can be nearly as effective as constant TET1 overexpression and more effective than JMJD2 constant overexpression. Moreover, the TET1–JMJD2 sequential transient overexpression with an initial overexpression level higher than that of constant TET1 or JMJD2 overexpression can show higher efficiency. This is practically relevant since cells may be able to tolerate higher overexpression levels if applied for a shorter duration. Overall, these results suggest that simpler reprogramming approaches, which involve the transient transfection of TET1 (and/or JMJD2) instead of genetically modifying the cells to obtain constant overexpression of TET1 (and/or JMJD2), could be a valuable option for enhancing reprogramming efficiency.

Another computational study was conducted with the aim of determining how the dosage of OCT4 overexpression affects the reprogramming process (Supplementary Note 2). The results suggest that higher levels of OCT4 improve the process efficiency and reduce latency variability. However, this conclusion is based on a single TF model of the pluripotency GRN, while it is well known that the pluripotency GRN includes and requires the reactivation of two additional TFs: Nanog and Sox2^32,62^. These TFs are linked to OCT4 through mutual activation interactions. Models of this multi-TF GRN have shown that multiple stable steady states can arise and that the pluripotent state does not necessarily correspond to the highest OCT4 level^63,64^ in accordance with experimental results in mouse^65^ and human^15,66^. It is therefore plausible that an intermediate overexpression level of OCT4 may be preferable to increase reprogramming efficiency in a model that can capture multiple stable steady states, each corresponding to different lineages. Thus, in order to use this model not only to study the OCT4 reactivation dynamics, but also the dynamics of the PL GRN, future studies will need to combine the OCT4-Nanog-Sox2 GRN model^63,67,68^, with the chromatin modification circuit model to enable concurrent investigation of the effect of TF overexpression dosage and chromatin state on the PL GRN dynamics.

Finally, in our model, dilution due to cell growth and division is captured by an effective decay reaction with first-order kinetics, which is one of the standard models used^69^. However, more elaborated models for dilution may be implemented to account for the cell cycle and the binomial partitioning of molecules at cell division^70,71^. We then conducted an additional computational study, described in Supplementary Note 4, in which we removed the first-order decay reactions and introduced the binomial partitioning of molecules at the end of the cell cycle. This study reveals that, while the time trajectories generated by an Epi OCT4 GRN model with binomial partitioning are less smooth compared to those from our original model, the trend with which TET1 and JMJD2 affect OCT4 reprogramming efficiency and latency variability does not significantly change (Supplementary Figs. 14 and 15). Furthermore, it has been shown that OCT4 gene transcription is affected by a dosage compensation effect^70^. This can be qualitatively captured by our model by varying the effective expression rate of the gene (i.e., ${\bar{p}}_{O}$). If ${\bar{p}}_{O}$ increases, due to imperfect compensation, then, a lower level of overexpression is required to achieve the same improvement in reprogramming efficiency (Supplementary Figs. 4, 5, 7, 9 and 13).

In conclusion, the model developed in this paper not only allows to mechanistically compare the effect of different reprogramming approaches on the OCT4 reactivation dynamics, but can also aid the rational design of new gene reactivation approaches and their application to cell fate reprogramming.

## Methods

All simulations in this paper were conducted using Gillespie’s Stochastic Simulation Algorithm (SSA)^43^.

### Reporting summary

Further information on research design is available in the Nature Research Reporting Summary linked to this article.

### Supplementary information

Supplementary Information file

Reporting summary

## Data Availability

MATLAB codes used to generate the graphs in the paper are available on a GitHub repository at https://github.com/simonbruno100/EpiOct4GRNpaper2023.

## References

[1] Graf T, Enver T (2009). Forcing cells to change lineages. Nature.

[2] Jopling, C., Boue, S. & Belmonte, J. Dedifferentiation, transdifferentiation and reprogramming: three routes to regeneration. *Nat. Rev. Mol. Cell Biol.* **12**, 79–89 (2011). 10.1038/nrm3043 21252997

[3] Hirschi K, Li S, Roy K (2014). Induced pluripotent stem cells for regenerative medicine. Annu. Rev. Biomed. Eng..

[4] Takahashi, K. & Yamanaka, S. Induction of pluripotent stem cells from mouse embryonic and adult fibroblast cultures by defined factors. *Cell* **126**, 663–676 (2006). 10.1016/j.cell.2006.07.024 16904174

[5] Takahashi, K. et al. Induction of pluripotent stem cells from adult human fibroblast by defined factors. *Cell* **131**, 861–872 (2007). 10.1016/j.cell.2007.11.019 18035408

[6] Malik N, Rao M (2013). A review of the methods for human iPSC derivation. Methods Mol. Biol..

[7] Goh, P. et al. A systematic evaluation of integration free reprogramming methods for deriving clinically relevant patient specific induced pluripotent stem (iPS) cells. *PLoS ONE* **8**, e81622 (2013). 10.1371/journal.pone.0081622 PMC3841145 24303062

[8] Schlaeger T (2015). A comparison of non-integrating reprogramming methods. Nat. Biotech..

[9] Takahashi K, Yamanaka S (2016). A decade of transcription factor-mediated reprogramming to pluripotency. Nat. Rev. Mol. Cell Biol..

[10] Theunissen T, Jaenisch R (2014). Molecular control of induced pluripotency. Cell Stem Cell.

[11] Carey B (2011). Reprogramming factor stoichiometry influences the epigenetic state and biological properties of induced pluripotent stem cells. Cell Stem Cell.

[12] Shu J (2013). Induction of pluripotency in mouse somatic cells with lineage specifiers. Cell.

[13] Warren L (2010). Highly efficient reprogramming to pluripotency and directed differentiation of human cells with synthetic modified mRNA. Cell Stem Cell.

[14] Heng B, Fussenegger M (2010). Integration-free reprogramming of human somatic cells to induced pluripotent stem cells (iPSCs) without viral vectors, recombinant DNA, and genetic modification. Methods Mol. Biol..

[15] Papapetrou E (2009). Stoichiometric and temporal requirements of oct4, sox2, klf4, and c-myc expression for efficient human iPSC induction and differentiation. PNAS.

[16] Tiemann U (2011). Optimal reprogramming factor stoichiometry increases colony numbers and affects molecular characteristics of murine induced pluripotent stem cells. Cytometry A.

[17] Meng X (2012). Efficient reprogramming of human cord blood cd34+ cells into induced pluripotent stem cells with oct4 and sox2 alone. Mol Ther..

[18] Nagamatsu G (2012). Optimal ratio of transcription factors for somatic cell reprogramming. J. Biol. Chem..

[19] Radzisheuskaya A (2013). A defined oct4 level governs cell state transitions of pluripotency entry and differentiation into all embryonic lineages. Nat. Cell Biol..

[20] Sui D (2014). Fine-tuning of iPSC derivation by an inducible reprogramming system at the protein level. Stem Cell Rep..

[21] Rais, Y. et al. Deterministic direct reprogramming of somatic cells to pluripotency. *Nature* **502**, 65–70 (2013). 10.1038/nature12587 24048479

[22] Chen, J. et al. H3k9 methylation is a barrier during somatic cell reprogramming into iPSCs. *Nat. Genet.* **45**, 34–42 (2013). 10.1038/ng.2491 23202127

[23] Yamaguch, S., Shen, L., Liu, Y., Sendler, D. & Zhang, Y. Role of tet1 in erasure of genomic imprinting. *Nature* **504**, 460–464 (2013). 10.1038/nature12805 PMC3957231 24291790

[24] Gao Y (2013). Replacement of oct4 by tet1 during iPSC induction reveals an important role of DNA methylation and hydroxymethylation in reprogramming. Cell Stem Cell.

[25] Huisinga K, Brower-Toland B, Elgin S (2006). The contradictory definitions of heterochromatin: transcription and silencing. Chromosoma.

[26] Allis, C., Caparros, M., Jenuwein, T. & Reinberg, D. *Epigenetics* 2nd edn (Cold Spring Harbor Laboratory Press, 2015).

[27] Meshorer E (2006). Hyperdynamic plasticity of chromatin proteins in pluripotent embryonic stem cells. Dev. Cell.

[28] Gaspar-Maia A, Alajem A, Meshorer E, Ramalho-Santos M (2011). Open chromatin in pluripotency and reprogramming. Nat. Rev. Mol. Cell Biol..

[29] Wang J, Jia S, Jia S (2016). New insights into the regulation of heterochromatin. Trends Genet..

[30] Penagos-Puig, A. & Furlan-Magaril, M. Heterochromatin as an important driver of genome organization. *Front. Cell Dev. Biol.* **8**, 579137 (2020). 10.3389/fcell.2020.579137 PMC7530337 33072761

[31] Luo, M. et al. Nurd blocks reprogramming of mouse somatic cells into pluripotent stem cells. *Stem Cells* **31**, 1278–1286 (2013). 10.1002/stem.1374 23533168

[32] Hanna, J. et al. Direct cell reprogramming is a stochastic process amenable to acceleration. *Nature* **462**, 595–601 (2009). 10.1038/nature08592 PMC2789972 19898493

[33] Boland M, Nazor K, Loring J (2014). Epigenetic regulation of pluripotency and differentiation. Circ. Res..

[34] Ludwig, A. et al. Binding of MBD proteins to DNA blocks Tet1 function thereby modulating transcriptional noise. *Nucleic Acid Res.* **45**, 2438–2457 (2017). 10.1093/nar/gkw1197 PMC5389475 27923996

[35] Radzisheuskaya A, Silva J (2014). Do all roads lead to oct4? The emerging concepts of induced pluripotency. Trends Cell Biol..

[36] Shi, G. & Jin, Y. Role of oct4 in maintaining and regaining stem cell pluripotency. *Stem Cell Res. Ther.* **1**, 39 (2010). 10.1186/scrt39 PMC3025441 21156086

[37] Hammachi F (2012). Transcriptional activation by oct4 is sufficient for the maintenance and induction of pluripotency. Cell Rep..

[38] Whetstine J (2006). Reversal of histone lysine trimethylation by the JMJD2 family of histone demethylases. Cell.

[39] Cloos P (2006). The putative oncogene GASC1 demethylates tri- and dimethylated lysine 9 on histone h3. Nature.

[40] Fodor BD (2006). Jmjd2b antagonizes h3k9 trimethylation at pericentric heterochromatin in mammalian cells. Genes Dev.

[41] Rasmussen, K. D. & Helin, K. Role of tet enzymes in DNA methylation, development, and cancer. *Genes Dev.* **30**, 733–750 (2016). 10.1101/gad.276568.115 PMC4826392 27036965

[42] Jin, C. et al. Tet1 is a maintenance DNA demethylase that prevents methylation spreading in differentiated cells. *Nucleic Acids Res.* **42**, 6956–6971 (2014). 10.1093/nar/gku372 PMC4066785 24875481

[43] Gillespie DT (2007). Stochastic simulation of chemical kinetics. Annu. Rev. Phys. Chem..

[44] Bruno S, Williams RJ, Del Vecchio D (2022). Epigenetic cell memory: the gene’s inner chromatin modification circuit. PLoS Comput. Biol..

[45] Artyomov, M. N., Meissner, A. & Chakraborty, A. K. A model for genetic and epigenetic regulatory networks identifies rare pathways for transcription factor induced pluripotency. *PLoS Comput. Biol*. **6**, e1000785 (2010). 10.1371/journal.pcbi.1000785 PMC2869311 20485562

[46] Flöttmann, M., Scharp, T. & Klipp, E. A stochastic model of epigenetic dynamics in somatic cell reprogramming. *Front. Physiol.* **3**, 216 (2012). 10.3389/fphys.2012.00216 PMC3384084 22754535

[47] Ashwin, S. S. & Sasai, M. Effects of collective histone state dynamics on epigenetic landscape and kinetics of cell reprogramming. *Nat. Rep.* **5**, 16746 (2015). 10.1038/srep16746 PMC4652167 26581803

[48] Chen T, Al-Radhawi M, Sontag E (2021). A mathematical model exhibiting the effect of DNA methylation on the stability boundary in cell-fate networks. Epigenetics.

[49] Smith ZD, Meissner A (2013). DNA methylation: roles in mammalian development. Nat. Rev. Genet..

[50] Athanasiadou, R. et al. Targeting of de novo DNA methylation throughout the oct-4 gene regulatory region in differentiating embryonic stem cells. *PLoS ONE* **5**, e9937 (2010). 10.1371/journal.pone.0009937 PMC2848578 20376339

[51] Huang, S., Litt, M. & Blakey, C. A. *Epigenetic Gene Expression and Regulation* (Academic Press, 2015).

[52] Ang, Y.-S. et al. Wdr5 mediates self-renewal and reprogramming via the embryonic stem cell core transcriptional network. *Cell* **145**, 183–197 (2011). 10.1016/j.cell.2011.03.003 PMC3097468 21477851

[53] Loh, Y.-H., Zhang, W., Chen, X., George, J. & Ng, H.-H. Jmjd1a and jmjd2c histone H3 Lys 9 demethylases regulate self-renewal in embryonic stem cells. *Genes Dev.* **21**, 2545–2557 (2007). 10.1101/gad.1588207 PMC2000320 17938240

[54] Wu, Y. et al. Oct4 and the small molecule inhibitor, sc1, regulates tet2 expression in mouse embryonic stem cells. *Mol. Biol. Rep.* **40**, 2897–2906 (2013). 10.1007/s11033-012-2305-5 23254757

[55] Zhang, T., Cooper, S. & Brockdorff, N. The interplay of histone modifications—writers that read. *EMBO Rep.* **16**, 1467–1481 (2015). 10.15252/embr.201540945 PMC4641500 26474904

[56] Kim, T. & Buratowski, S. Dimethylation of h3k4 by set1 recruits the set3 histone deacetylase complex to 50 transcribed regions. *Cell* **137**, 259–272 (2009). 10.1016/j.cell.2009.02.045 PMC2802783 19379692

[57] Brennera, C. et al. Myc represses transcription through recruitment of DNA methyltransferase corepressor. *EMBO J.* **24**, 336–346 (2005). 10.1038/sj.emboj.7600509 PMC545804 15616584

[58] Khoueiry R (2017). Lineage-specific functions of tet1 in the postimplantation mouse embryo. Nat. Genet..

[59] KP K (2011). Tet1 and tet2 regulate 5-hydroxymethylcytosine production and cell lineage specification in mouse embryonic stem cells. Cell Stem Cell.

[60] von Meyenn, F. et al. Impairment of DNA methylation maintenance is the main cause of global demethylation in naive embryonic stem cells. *Mol. Cell* **62**, 848–861 (2016). 10.1016/j.molcel.2016.04.025 PMC4914828 27237052

[61] Chen, T. & Dent, S. Y. R. Chromatin modifiers: regulators of cellular differentiation. *Nat. Rev. Genet.* **15**, 93–106 (2014). 10.1038/nrg3607 PMC3999985 24366184

[62] Wernig M (2007). In vitro reprogramming of fibroblasts into a pluripotent ES-cell-like state. Nature.

[63] Del Vecchio, D., Abdallah, H., Qian, Y. & Collins, J. J. A blueprint for a synthetic genetic feedback controller to reprogram cell fate. *Cell Syst.* **4**, 109–120.e11 (2017). 10.1016/j.cels.2016.12.001 PMC5326680 28065574

[64] Bruno, S., Al-Radhawi, M., Sontag, E. D. & Del Vecchio, D. Stochastic analysis of genetic feedback controllers to reprogram a pluripotency gene regulatory network. in *Proc of American Control Conference* 5089–5096 (2019). 10.23919/acc.2019.8814355 PMC7043063 32103851

[65] Niwa H, Miyazaki JI, Smith AG (2000). Quantitative expression of oct-3/4 defines differentiation, dedifferentiation or self-renewal of es cells. Nat. Genet..

[66] Wang Z, Oron E, Nelson B, Razis S, Ivanova N (2012). Distinct lineage specification roles for nanog, oct4, and sox2 in human embryonic stem cells. Cell Stem Cell.

[67] Boyer LA (2005). Core transcriptional regulatory circuitry in human embryonic stem cells. Cell.

[68] Jaenisch R, Young R (2008). Stem cells, the molecular circuitry of pluripotency and nuclear reprogramming. Cell.

[69] Friedman, N., Cai, L. & Xie, X. S. Linking stochastic dynamics to population distribution: an analytical framework of gene expression. *Phys. Rev. Lett.* **97**, 168302 (2006). 10.1103/PhysRevLett.97.168302 17155441

[70] Skinner, S. O. et al. Single-cell analysis of transcription kinetics across the cell cycle. *Elife* **5**, e12175 (2016). 10.7554/eLife.12175 PMC4801054 26824388

[71] Beentjes, C. H. L., Perez-Carrasco, R. & Grima, R. Exact solution of stochastic gene expression models with bursting, cell cycle and replication dynamics. *Phys. Rev. E* **101**, 032403 (2020). 10.1103/PhysRevE.101.032403 32290003

