# Supplementary material for: Epigenetic OCT4 regulatory network: stochastic analysis of cellular reprogramming
Source: NPJ Syst Biol Appl. 2024 Jan 6;10:3. doi: 10.1038/s41540-023-00326-0 (PMC10771499; doi:10.1038/s41540-023-00326-0)
Supplement: Supplementary file 1 — Supplementary Information file [file 41540_2023_326_MOESM1_ESM.pdf]

Epigenetic OCT4 regulatory network: stochastic analysis of cellular reprogramming

Simone Bruno[1], Thorsten M. Schlaeger[2] and Domitilla Del Vecchio[1,*]

[1]*Department of Mechanical Engineering, Massachusetts Institute of Technology, 77 Massachusetts Avenue, Cambridge, MA 02139, USA.*

[2]*Boston Children's Hospital Stem Cell Program, Boston Children's Hospital, 300 Longwood Avenue, Boston, MA 02115, USA.*

[*]*Corresponding Author. Email:* ddv@mit.edu

Supplementary information

1 Relationship between the time t and the normalized time τ

Let $\mathrm{D_{tot}}$ denote the total number of modifiable nucleosomes within the gene of interest, Ω represent the reaction volume, and k_M^A be the rate of the auto-catalytic reaction through which activating histone modifications enhances the establishment of modifications of the same kind to nearby nucleosomes. We define the normalize time τ as $\tau = t\frac{k_M^A}{\Omega}\mathrm{D_{tot}}$. Considering the assumption that there is approximately one nucleosome for every 200 base pairs ([1], Chapter 4), and taking into account that an average gene spans $10 \times 10^3 - 20 \times 10^3$ base pairs [2], the average value of $\mathrm{D_{tot}}$ can be considered to range between 50 and 100. Specifically, in all simulations, we assume $\mathrm{D_{tot}}$ to be 50. Furthermore, in our simulations we also assume $k_M^A/\Omega = 0.008\ \mathrm{h}^{-1}$.

Then, an interval of time of $t = 21$ days $= 504$ h corresponds to $\tau = 504 \cdot 0.008 \cdot 50 = 201.6$.

2 Reprogramming based on different levels of OCT4 overexpression

Here, we conducted several simulations using SSA to analyze the trajectory of the active chromatin state of the OCT4 gene, n_O^A, starting from a fully repressed state ($n_O^A = n_T^A = n_J^A = 0$), for different levels of OCT4 overexpression, that is, $\bar{u}_O > 0$ (Figure 2).

The results of this analysis are shown in Supplementary Figure 2. For low values of $\bar{u}_0$, none of the trajectories reach the active state in the time span observed, corresponding to 21 days. Then, by increasing $\bar{u}_O$ some trajectories reach the OCT4 gene active state, but the latency variability does not seem to be reduced by increasing the overexpression level of OCT4 (the input $\bar{u}_O$) among the trajectories.

These results indicates that the higher the OCT4 level is, the more efficient the OCT4 reactivation process is.

3 Reprogramming approaches based on constant OCT4 overexpression and transient overexpression of TET1 and/or JMJD2

Here, for each epigenetic modifier TET1 and JMJD2, we compared the efficiency $\%O^A$ of the two reprogramming approaches, in which, together with OCT4 constant overexpression, the epigenetic modifier is either constantly or transiently overexpressed. Let us start from the case in which TET1 is overexpressed and let us define the initial level of TET1 overexpresssion as $\bar{u}_T^0$. Then, we modeled a constant overexpression of TET1 as $\bar{u}_T = \bar{u}_T^0$, as done in the "Results" section, while we modeled a transient TET1 overexpression as

$$\bar{u}_T = \bar{u}_T^0 e^{-\varepsilon_d \tau}, \tag{1}$$

where ε_d is the normalized dilution rate constant.

Similarly, defining the initial level of JMJD2 overexpresssion as $\bar{u}_J^0$, we modeled a constant overexpression of JMJD2 as $\bar{u}_J = \bar{u}_J^0$, while we modeled a transient JMJD2 overexpression as $\bar{u}_J = \bar{u}_J^0 e^{-\varepsilon_d \tau}$.

The results of this computational analysis show that the transient overexpression of TET1 or JMJD2, although less effective than constant overexpression, enhances the efficiency of the OCT4 reactivation process (Supplementary Figure 7 and Supplementary Figure 9). Additionally, the effectiveness of transient overexpression of epigenetic modifiers becomes more comparable to constant overexpression when the initial level of overexpression is higher (Supplementary Figure 8 and Supplementary Figure 10).

We then analyzed the effect of sequential, transient overexpression of TET1 and JMJD2. Specifically, we studied the sequential process in which we first introduce TET1 and then JMJD2. This is because, in practical scenarios, we are likely to encounter a parameter regime where the enhancement of DNA methylation establishment by H3K9me3 is not highly effective, that is low r (see "Results" section). Since r is low, adding JMJD2 to erase repressive histone modifications, and, with them, the enhancement of DNA methylation establishment, is not effective unless DNA methylation is quickly erased. A fast erasure of DNA methylation can be achieved by adding TET1. Our computational analysis reveals that when the initial level of transient overexpression for both TET1 and JMJD2 is sufficiently high and matches the level of constant overexpression for either TET1 or JMJD2, the sequential transient overexpression of TET1 followed by JMJD2 can be nearly as effective as constant TET1 overexpression and more effective than JMJD2 constant overexpression (Supplementary Figure 11, right-hand side plot). Moreover, when comparing the TET1-JMJD2 sequential transient overexpression with an initial overexpression level of both JMJD2 and TET1 that is higher compared to the initial level of constant TET1 or JMJD2 overexpression (Supplementary Figure 12), TET1 - JMJD2 sequential, transient overexpression can show higher efficiency compared to both constant TET1 overexpression and constant JMJD2 overexpression. This is practically relevant because a high overexpression level that is not tolerated by the cells for extended amount of time, as in constitutive overexpression, may be well tolerated if lasting only a short amount of time.

4 Computational analysis by introducing in the model the binomial partitioning of molecules at the end of the cell cycle

In our current model, we describe the effect of dilution due to cell growth and division as an effective decay reaction with first-order kinetics, which is one of the standard models used [3]. However, different models may be implemented to more accurately incorporate the cell cycle into the dilution

process. Here, we develop a more elaborated model in which, instead of introducing the dilution of a generic species X as a 1st order reaction $X \rightarrow \emptyset$, with rate constant $\delta > 0$, we explicitly introduce the growth rate and division of cells over time and then the binomial partitioning of molecules at the end of the cell cycle. Then, we study the new model by using the Adiabatic Time-Dependent Gillespie (ATG) approach [4]. Compared to the standard Gillespie algorithm used for our original model, in the ATG the volume is not constant and the propensity functions that depend on the volume become a function of time. More precisely, we assume that the volume grows exponentially with time until it doubles and divides. The key steps of the ATC algorithm can be summarized as follows: let us assume that, within the volume $\Omega(t)$, $\mathrm{X_1, ..., X_n}$ species interact through reactions $R_1, ...R_m$. Furthermore, let us introduce the state vector $x = [x_1, x_2, ..., x_j, ..., x_n]$, in which x_j corresponds to the molecular count of species $\mathrm{X_j}$, $j = 1, ..., n$. Then, the propensity function of reaction R_i, $i = 1, ..., m$, that is, the probability that reaction R_i occurs in the time interval $(t, t + d\tau]$, can be written as $a_i(x, t)d\tau$, and the change in the molecular count associated to R_i can be written as Δ_i.

Now,

- **Step A)** Set $t = 0$ and initial values for $x_1, ..., x_n$.
- **Step B)** Set the initial volume $\Omega(t) = \Omega(0)$, the growth rate $\delta > 0$ and the doubling time $T = ln(2)/\delta$.
- **Step C)** Initialize the number of cell division, c, to 0, i.e., $c \leftarrow 0$.
- **Step D)** Introduce the volume temporal evolution, i.e., $\Omega(t) = \Omega_0 e^{[\delta(t-cT)]}$, and compute the propensity functions $a_i(x, t)$, $i = 1, ..., m$.
- **Step E)** Generate two random numbers $r_1, r_2 \in [0, 1)$.
- **Step F)** Compute τ, that is, the time interval until the next reaction, by using the formula $\tau = -\frac{ln(r_1)}{\sum_{i=1}^{M} a_i(x,t)}$.
- **Step G)** Find the next reaction R_i that will occur by taking i to be the positive integer such that $\sum_{k=1}^{i-1} a_k < r_2 \sum_{i=1}^{m} a_i \leq \sum_{k=1}^{i} a_k$.
- **Step H)** If $t + \tau \leq (c + 1)T$, then set $x(t + \tau) \leftarrow x(t) + \Delta_i$. If $t + \tau > (c + 1)T$, then set $c \leftarrow c + 1$, $t \leftarrow cT$, $x \leftarrow binornd(x, 0.5)$, $\Omega \leftarrow \Omega_0$. Restart from **Step D**.

For the simulations, we considered all the reactions listed in Figure 1b of the paper, except for the 1st order reactions modeling dilution, which we removed. As for the reactions with a rate constant $\delta' > 0$, which represent the effective passive erasure of DNA methylation resulting from the balance between dilution and the maintenance process through DNMT1, we also removed them. Instead, we introduced two additional reactions ($\mathrm{D} \rightarrow \mathrm{D_1^R}$ and $\mathrm{D_2^R} \rightarrow \mathrm{D_{12}^R}$) with a rate constant of $k_M^{DNMT1} > 0$ to keep in our model the DNA methylation maintenance process via DNMT1.

Our computational study shows that, while the time trajectories obtained with the new model are less smooth compared to the ones obtained with our original model, the trend with which the overexpression of TET1 and JMJD2 affect the OCT4 reprogramming efficiency and latency variability is not significantly affected (Supplementary Figure 14 and Supplementary Figure 15).

5 Supplementary Figures

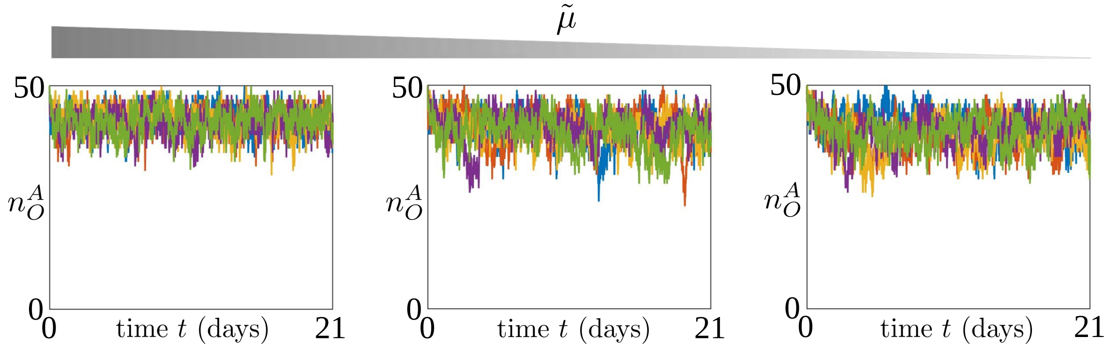

Supplementary Figure 1: **H3K9me3 erasure rate does not have a relevant impact on the dynamics of OCT4 during differentiation.** Time trajectories of n_O^A (total amount of nucleosomes modified with activating chromatin modifications for the OCT4 gene) starting from the OCT4 fully active state for different values of $\tilde{\mu}$. In all plots, on the x axis we have the time (days). The parameter values used for these simulations can be found in Supplementary table 2. In particular, we set $\tilde{\mu} = 1, 0.15, 0.07$, $\varepsilon_d = 0.3$, $\bar{p}_O = \bar{p}_T = \bar{p}_J = 3.2$, $\eta = 0.1$, $\tilde{\mu}' = 1$, $\varepsilon_e = 0.3$ and $\varepsilon' = 1$. In our model, parameter $\tilde{\mu}$ quantifies the asymmetry between the erasure rates of repressive histone modifications and activating histone modifications. Mathematical definition of $\tilde{\mu}$ can be found in Eq. (2). For all simulations, we implemented the reactions listed in Figure 1 with the SSA [5] and we considered a time span of 21 days ($\tau = 201.6$) and $D_{\text{tot}} = 50$ (see Supplementary Note 1).

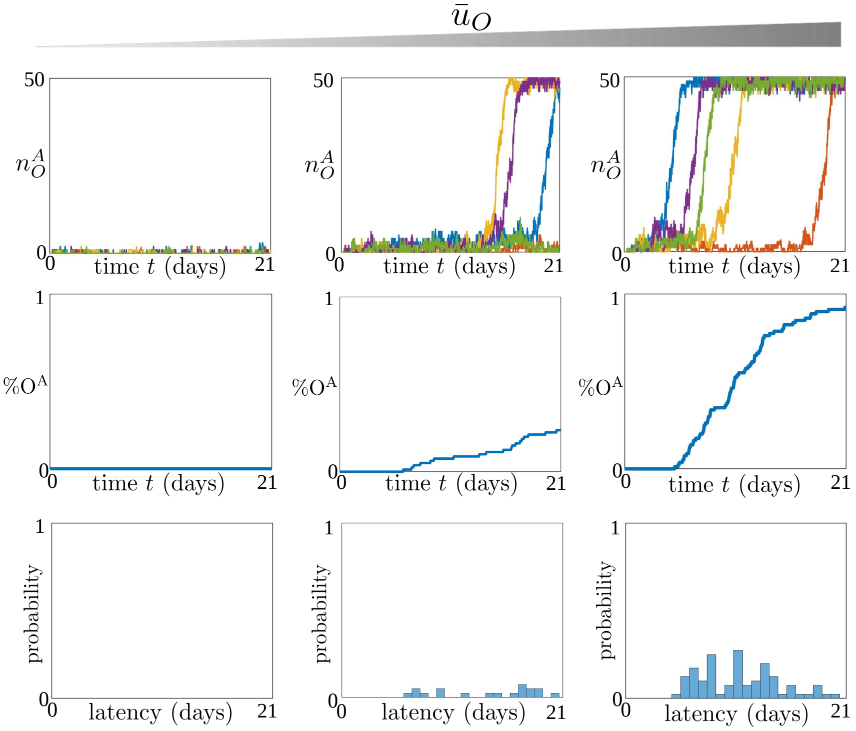

Supplementary Figure 2: **Higher levels of OCT4 overexpression increase the efficiency of the reprogramming process.** Upper plots: time trajectories of n_O^A (total amount of nucleosomes modified with activating chromatin modifications for the OCT4 gene) starting from the OCT4 fully repressed state ($n_O^A = 0$) for different values of $\bar{u}_O$. Intermediate plots: %O$^{\rm A}$, that is, the normalized amount of $N = 100$ time trajectories which reach $n_O^A \geq 40$, starting from $n_O^A = 0$. Lower plots: histogram plot showing the probability of a specific latency value across all the $N = 100$ simulations. In all plots, on the x axis we have the time normalized with respect to $k_M^A D_{tot}$, $\tau = t k_M^A D_{tot}$. The parameter values used for these simulations can be found in Supplementary table 2. In particular, we consider three values of $\bar{u}_O$ (i.e., $\bar{u}_O = 160, 220, 320$), and we set $\bar{u}_T = 0$, $\bar{u}_J = 0$, $\tilde{\mu}' = 0.5$, $\varepsilon_d = 0.2$, $\varepsilon_e = 0.2$, $\bar{p}_O = \bar{p}_T = \bar{p}_J = 5$, $\eta = 0.1$, $\tilde{\mu} = 1$ and $\varepsilon' = 1$ (See Supplementary Figure 13 for how the parameters $\tilde{\mu}'$ and $\bar{p}$ influence the impact of $\bar{u}_O$ on the reprogramming process). For all simulations, we implemented the reactions listed in Figure 1 with the SSA [5] and we considered a time span of 21 days ($\tau = 201.6$) and $\rm D_{tot} = 50$ (see Supplementary Note 1).

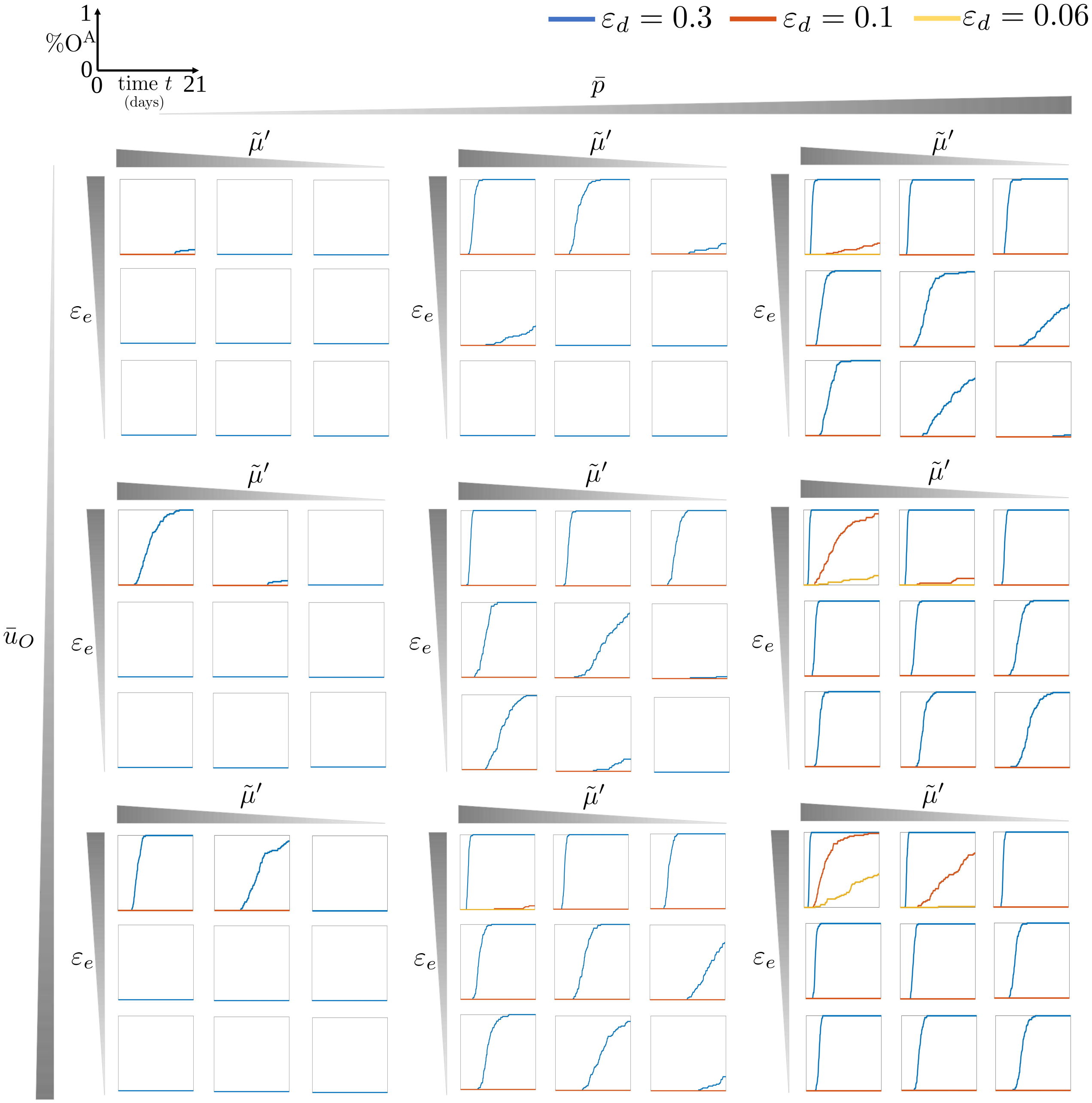

Supplementary Figure 3: **Effect of the parameter ε_d on efficiency and latency variability of reprogramming through overexpression of OCT4 for different values of $\bar{u}_O$, ε_e, $\tilde{\mu}'$, and $\bar{p}$.** %O^{A}, that is, the normalized amount of $N = 100$ time trajectories which reach $n_O^A \geq 40$, starting from $n_O^A = 0$, for different values of ε_d. In all plots, on the x axis we have the time (days). The parameter values used for these simulations can be found in Supplementary table 3. In particular, we consider three values of ε_d ($\varepsilon_d = 0.3, 0.1, 0.06$), three values of $\tilde{\mu}'$ ($\tilde{\mu}' = 1, 0.5, 0.2$), three values of ε_e ($\varepsilon_e = 0.3, 0.1, 0.06$), three values of $\bar{p}$ ($\bar{p} = 1, 2.5, 5$), in which $\bar{p} = \bar{p}_O = \bar{p}_T = \bar{p}_J$, three values of $\bar{u}_O$ ($\bar{u}_O = 160, 320, 480$), and we set $\eta = 0.1$, $\tilde{\mu} = 1$ and $\varepsilon' = 1$. Definitions of the parameters $\bar{u}_O$, ε_d, ε_e, $\tilde{\mu}'$, $\bar{p}_O$, $\bar{p}_J$, and $\bar{p}_T$ is given in the "Model of the epigenetic OCT4 gene regulatory network" subsection. For all simulations, we implemented the reactions listed in Figure 1 with the SSA [5], we considered a time span of 21 days ($\tau = 201.6$), and $\text{D}_{\text{tot}} = 50$ (see Supplementary Note 1).

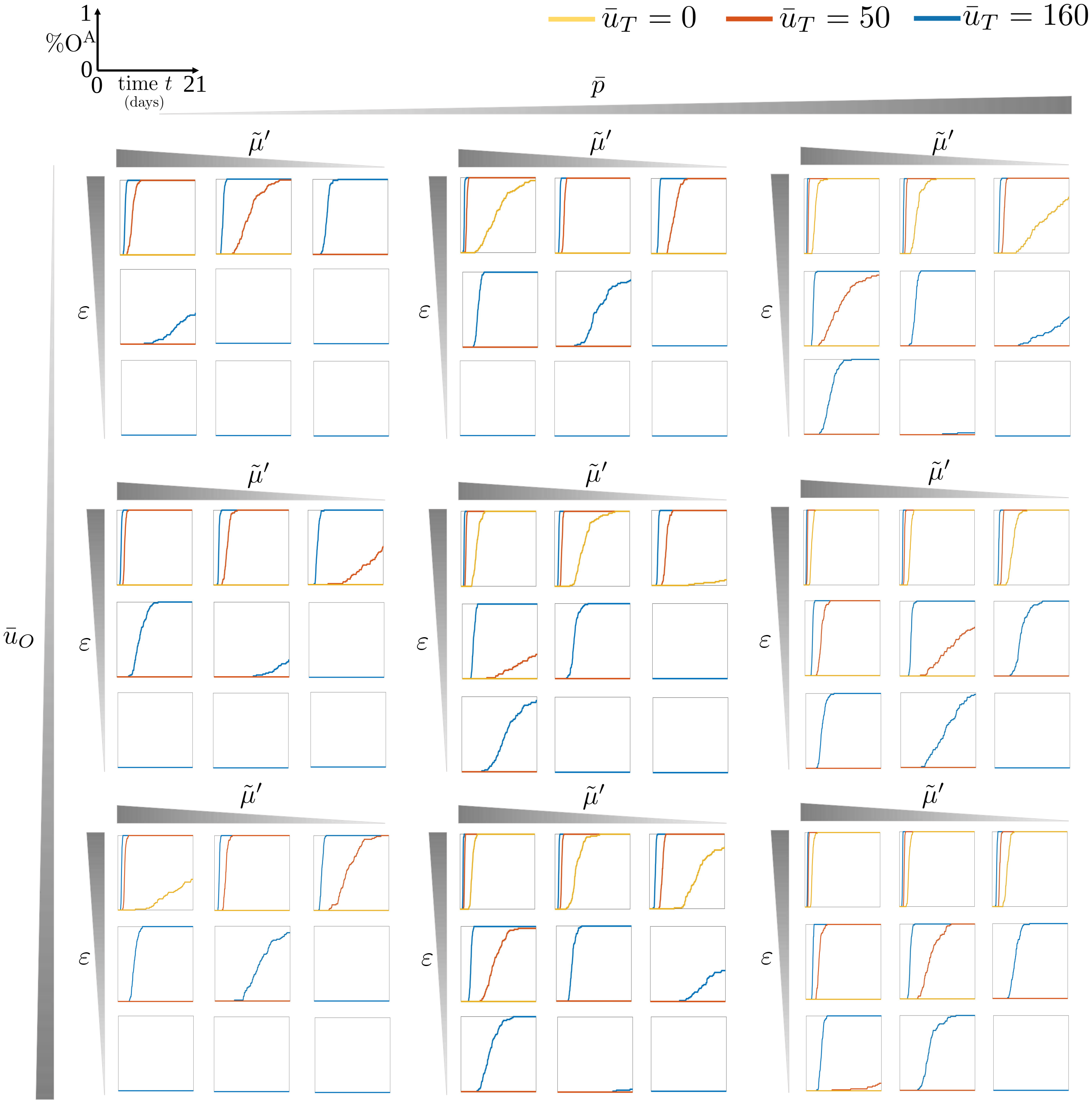

Supplementary Figure 4: **Effect of TET1 overexpression ($\bar{u}_T$) on efficiency and latency variability of reprogramming through overexpression of OCT4 for different values of $\bar{u}_O$, ε_d, ε_e, $\tilde{\mu}'$, and $\bar{p}$.** %O^A, that is, the normalized amount of $N = 100$ time trajectories which reach $n_O^A \geq 40$, starting from $n_O^A = 0$, for different values of $\bar{u}_T$. In all plots, on the x axis we have the time (days). The parameter values used for these simulations can be found in Supplementary table 3. In particular, we consider three values of $\bar{u}_T$ ($\bar{u}_T = 0, 50, 160$), three values of $\tilde{\mu}'$ ($\tilde{\mu}' = 1, 0.5, 0.2$), three values of ε ($\varepsilon = 0.3, 0.1, 0.06$), in which $\varepsilon = \varepsilon_d = \varepsilon_e$, three values of $\bar{p}$ ($\bar{p} = 1, 2.5, 5$), in which $\bar{p} = \bar{p}_O = \bar{p}_T = \bar{p}_J$, three values of $\bar{u}_O$ ($\bar{u}_O = 160, 320, 480$), and we set $\eta = 0.1$, $\tilde{\mu} = 1$ and $\varepsilon' = 1$. Definitions of the parameters $\bar{u}_O$, $\bar{u}_T$, ε_d, ε_e, $\tilde{\mu}'$, $\bar{p}_O$, $\bar{p}_J$, and $\bar{p}_T$ is given in the "Model of the epigenetic OCT4 gene regulatory network" subsection. For all simulations, we implemented the reactions listed in Figure 1 with the SSA [5], we considered a time span of 21 days ($\tau = 201.6$), and $D_{tot} = 50$ (see Supplementary Note 1).

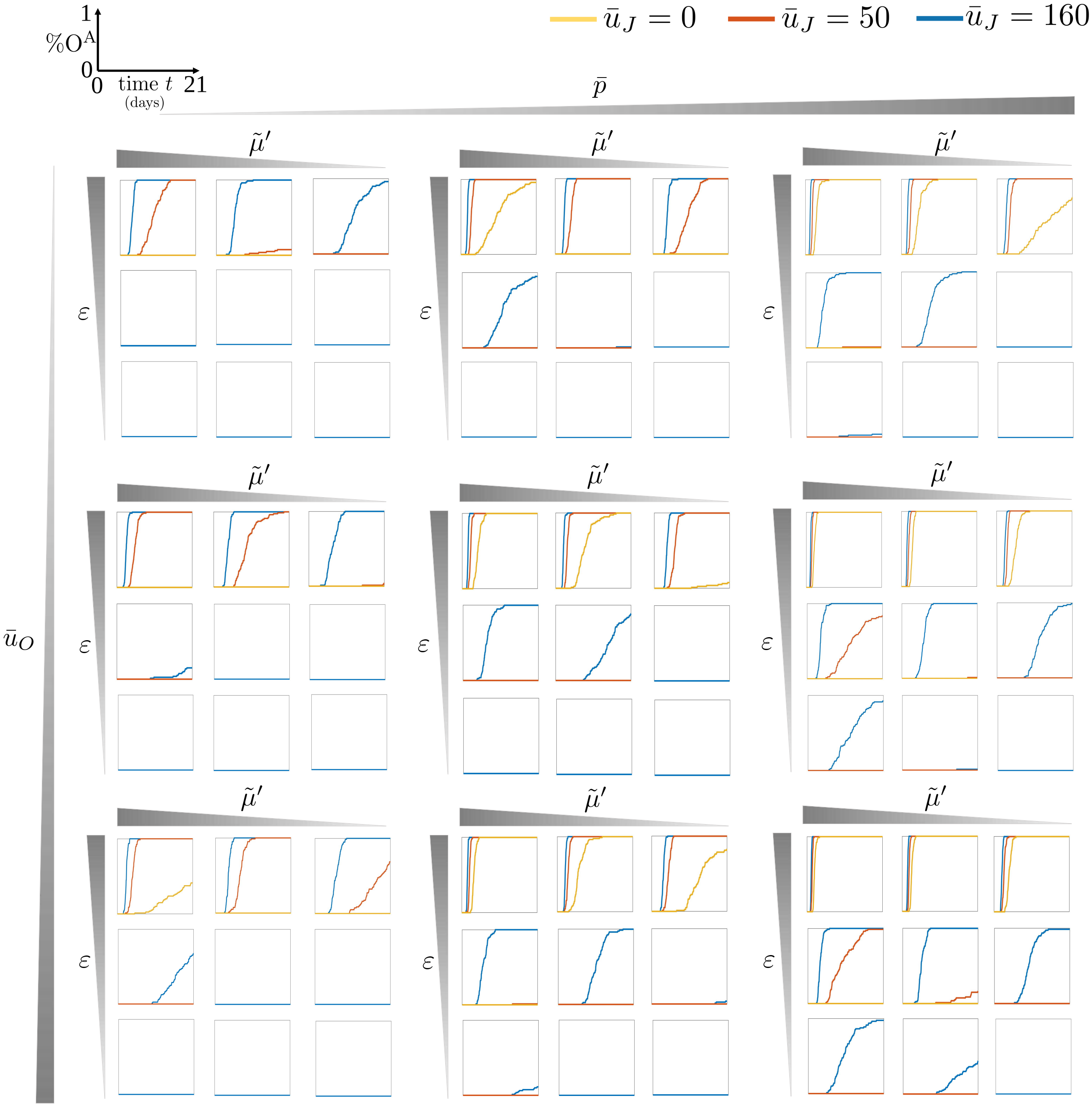

Supplementary Figure 5: **Effect of JMJD2 overexpression ($\bar{u}_J$) on efficiency and latency variability of reprogramming through overexpression of OCT4 for different values of $\bar{u}_O$, ε_d, ε_e, $\tilde{\mu}'$, and $\bar{p}$.** %O^A, that is, the normalized amount of $N = 100$ time trajectories which reach $n_O^A \geq 40$, starting from $n_O^A = 0$, for different values of $\bar{u}_J$. In all plots, on the x axis we have the time (days). The parameter values used for these simulations can be found in Supplementary table 3. In particular, we consider three values of $\bar{u}_J$ ($\bar{u}_J = 0, 50, 160$), three values of $\tilde{\mu}'$ ($\tilde{\mu}' = 1, 0.5, 0.2$), three values of ε ($\varepsilon = 0.3, 0.1, 0.06$), in which $\varepsilon = \varepsilon_d = \varepsilon_e$, three values of $\bar{p}$ ($\bar{p} = 1, 2.5, 5$), in which $\bar{p} = \bar{p}_O = \bar{p}_T = \bar{p}_J$, three values of $\bar{u}_O$ ($\bar{u}_O = 160, 320, 480$), and we set $\eta = 0.1$, $\tilde{\mu} = 1$ and $\varepsilon' = 1$. Definitions of the parameters $\bar{u}_O$, $\bar{u}_J$, ε_d, ε_e, $\tilde{\mu}'$, $\bar{p}_O$, $\bar{p}_J$, and $\bar{p}_T$ is given in the "Model of the epigenetic OCT4 gene regulatory network" subsection. For all simulations, we implemented the reactions listed in Figure 1 with the SSA [5], we considered a time span of 21 days ($\tau = 201.6$), and $\mathrm{D_{tot}} = 50$ (see Supplementary Note 1).

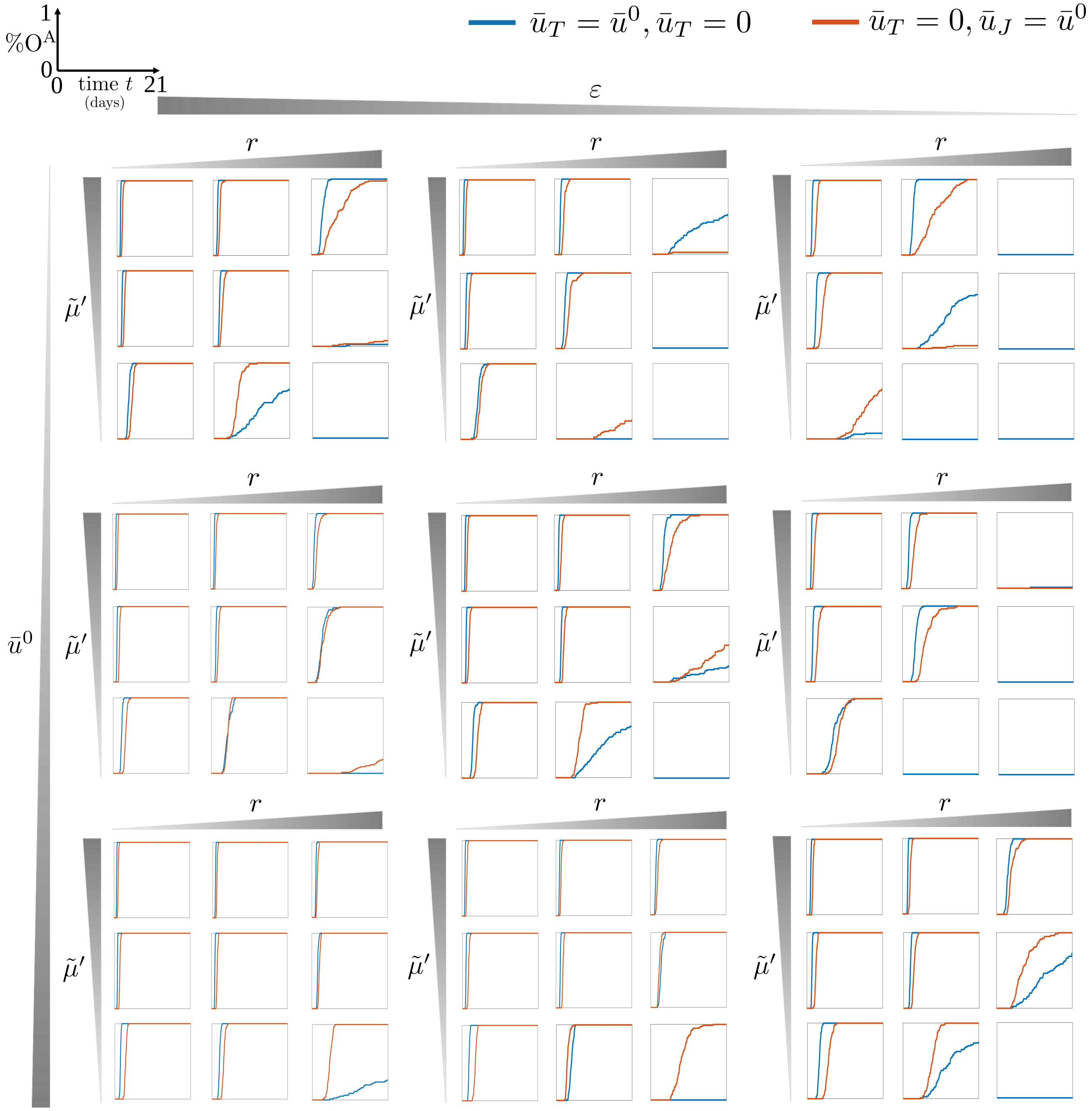

Supplementary Figure 6: **Comparison of the effect of JMJD2 overexpression ($\bar{u}_J$) and TET1 overexpression ($\bar{u}_T$) on efficiency and latency variability of reprogramming through overexpression of OCT4 for different values of r and μ'.** %O^{A}, that is, the normalized amount of $N = 100$ time trajectories which reach $n_O^A \geq 40$, starting from $n_O^A = 0$, for different values of r and μ'. In all plots, on the x axis we have the time (days). The parameter values used for these simulations can be found in Supplementary table 4. In particular, we consider three values of r ($r = 5, 1, 0.2$), three values of $\tilde{\mu}'$ ($\tilde{\mu}' = 1, 0.5, 0.1$), three values of $\bar{u}^0$ ($\bar{u}^0 = 100, 160, 320$), in which either $\bar{u}_T = \bar{u}^0$ (blue lines) or $\bar{u}_J = \bar{u}^0$ (red lines), three values of ε ($\varepsilon = 0.3, 0.1, 0.06$), in which $\varepsilon = \varepsilon_d = \varepsilon_e$, and we set $\bar{u}_O = 160$, $\bar{p} = 5$, $\eta = 0.1$, $\tilde{\mu} = 1$ and $\varepsilon' = 1$. Definitions of the parameters $\bar{u}_O$, $\bar{u}_T$, $\bar{u}_J$, r and $\tilde{\mu}'$ is given in the "Model of the epigenetic OCT4 gene regulatory network" subsection. For all simulations, we implemented the reactions listed in Figure 1 with the SSA [5], we considered a time span of 21 days ($\tau = 201.6$), and $\mathrm{D_{tot}} = 50$ (see Supplementary Note 1).

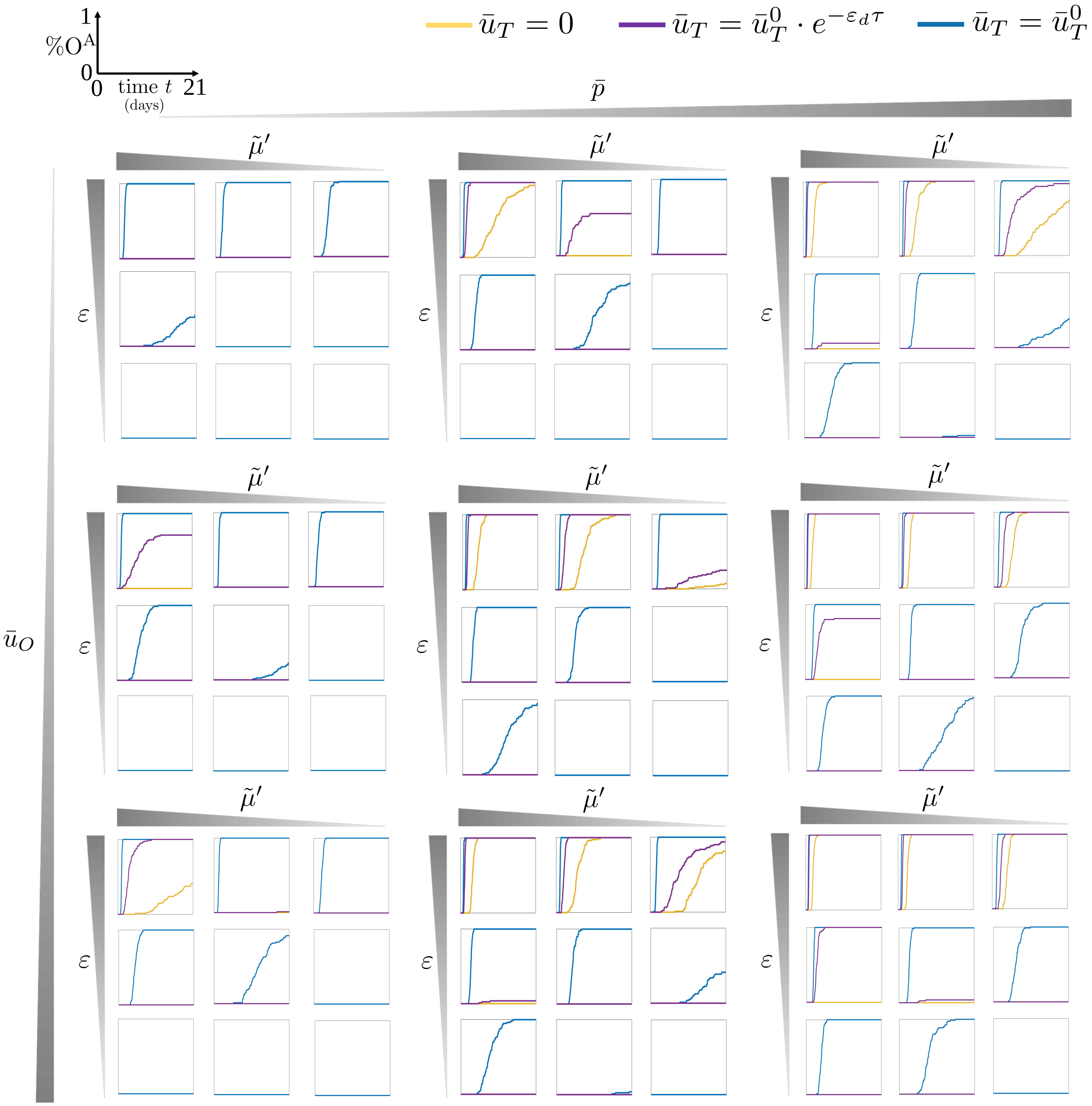

Supplementary Figure 7: **Comparison of the effect of constant and transient TET1 overexpression on efficiency and latency variability of reprogramming through overexpression of OCT4 for different values of $\bar{u}_O$, ε_d, ε_e, $\tilde{\mu}'$, and $\bar{p}$.** $\%\mathrm{O}^\mathrm{A}$, that is, the normalized amount of $N = 100$ time trajectories which reach $n_O^A \geq 40$, starting from $n_O^A = 0$, for different parameter values. In all plots, on the x axis we have the time (days). The parameter values used for these simulations can be found in Supplementary table 5. In particular, we consider three values of $\tilde{\mu}'$ ($\tilde{\mu}' = 1, 0.5, 0.2$), three values of ε ($\varepsilon = 0.3, 0.1, 0.06$), in which $\varepsilon = \varepsilon_d = \varepsilon_e$, three values of $\bar{p}$ ($\bar{p} = 1, 2.5, 5$), in which $\bar{p} = \bar{p}_O = \bar{p}_T = \bar{p}_J$, three values of $\bar{u}_O$ ($\bar{u}_O = 160, 320, 480$), and we set $\bar{u}_T^0 = 160$, $\eta = 0.1$, $\tilde{\mu} = 1$ and $\varepsilon' = 1$. In all the plots, we represented $\%\mathrm{O}^\mathrm{A}$ for the case in which only OCT4 is overexpressed (yellow curve). Definitions of the parameters $\bar{u}_O$, $\bar{u}_T$, ε_d, ε_e, $\tilde{\mu}'$, $\bar{p}_O$, $\bar{p}_J$, and $\bar{p}_T$ is given in the "Model of the epigenetic OCT4 gene regulatory network" subsection. For all simulations, we implemented the reactions listed in Figure 1 with the SSA [5], we considered a time span of 21 days ($\tau = 201.6$), and $\mathrm{D_{tot}} = 50$ (see Supplementary Note 1).

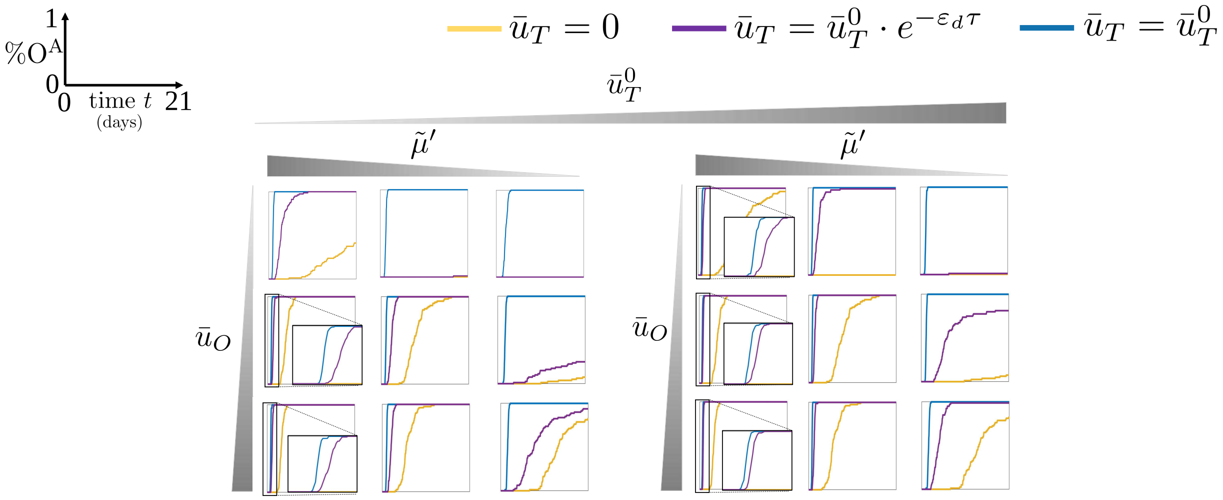

Supplementary Figure 8: **The effectiveness of transient overexpression of TET1 becomes more comparable to constant overexpression when the initial level of overexpression is higher.** %O^A, that is, the normalized amount of $N = 100$ time trajectories which reach $n_O^A \geq 40$, starting from $n_O^A = 0$, for different values of $\bar{u}_T^0$. In all plots, on the x axis we have the time (days). The parameter values used for these simulations can be found in Supplementary table 6. In particular, we consider two values of $\bar{u}_T^0$ ($\bar{u}_T^0 = 160, 320$), three values of $\tilde{\mu}'$ ($\tilde{\mu}' = 1, 0.5, 0.2$), three values of $\bar{u}_O$ ($\bar{u}_O = 160, 320, 480$), and we set $\varepsilon = 0.3$, in which $\varepsilon = \varepsilon_d = \varepsilon_e$, $\bar{p} = 2.5$, in which $\bar{p} = \bar{p}_O = \bar{p}_T = \bar{p}_J$, $\eta = 0.1$, $\tilde{\mu} = 1$ and $\varepsilon' = 1$. In all the plots, we represented %O^A for the case in which only OCT4 is overexpressed (yellow curve). For all simulations, we implemented the reactions listed in Figure 1 with the SSA [5], we considered a time span of 21 days ($\tau = 201.6$), and $D_{tot} = 50$ (see Supplementary Note 1).

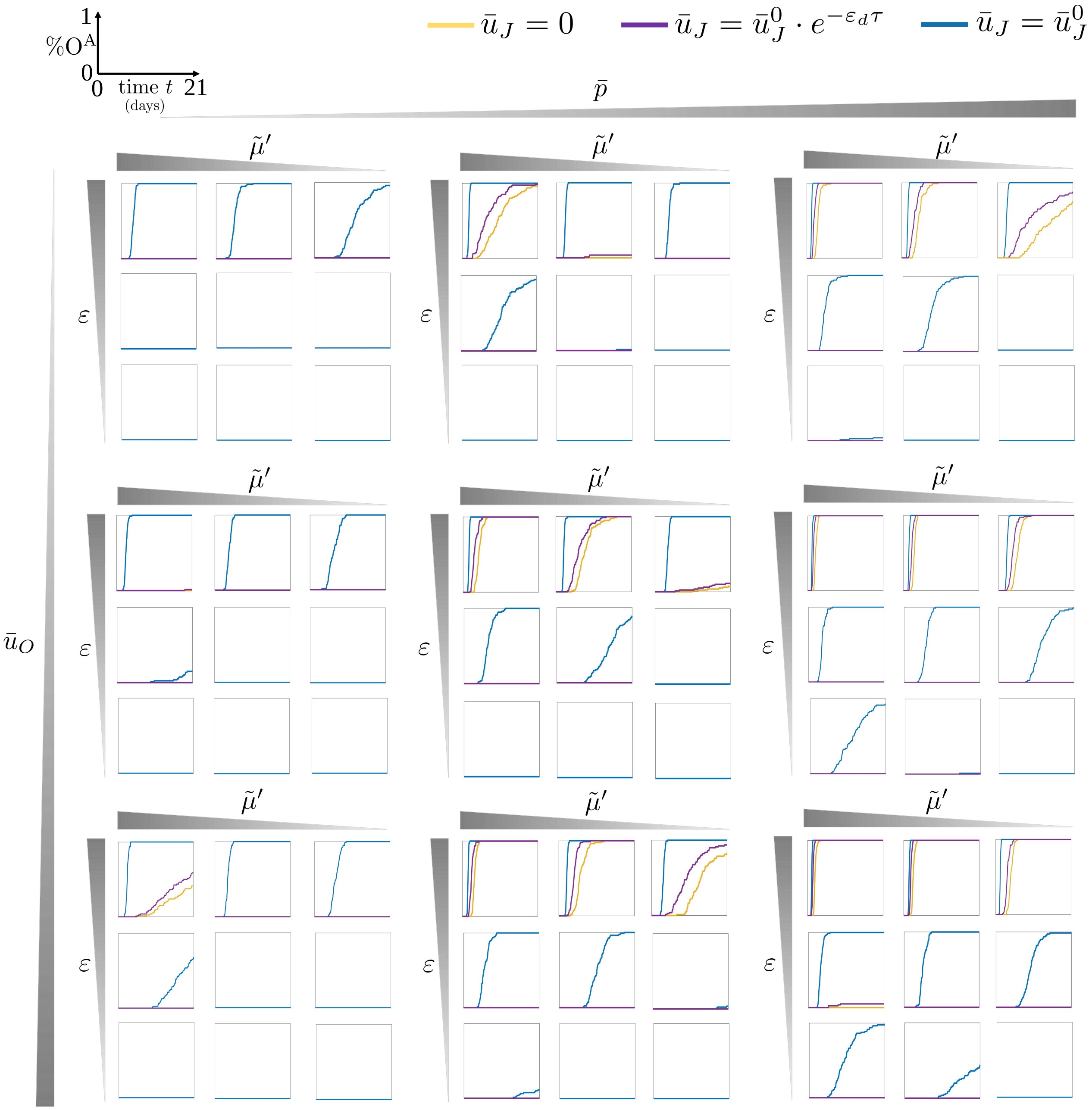

Supplementary Figure 9: **Comparison of the effect of constant and transient JMJD2 overexpression on efficiency and latency variability of reprogramming through overexpression of OCT4 for different values of $\bar{u}_O$, ε_d, ε_e, $\tilde{\mu}'$, and $\bar{p}$.** %O^{A}, that is, the normalized amount of $N = 100$ time trajectories which reach $n_O^A \geq 40$, starting from $n_O^A = 0$, for different parameter values. In all plots, on the x axis we have the time (days). The parameter values used for these simulations can be found in Supplementary table 5. In particular, we consider three values of $\tilde{\mu}'$ ($\tilde{\mu}' = 1, 0.5, 0.2$), three values of ε ($\varepsilon = 0.3, 0.1, 0.06$), in which $\varepsilon = \varepsilon_d = \varepsilon_e$, three values of $\bar{p}$ ($\bar{p} = 1, 2.5, 5$), in which $\bar{p} = \bar{p}_O = \bar{p}_T = \bar{p}_J$, and three values of $\bar{u}_O$ ($\bar{u}_O = 160, 320, 480$), and we set $\bar{u}_J^0 = 160$, $\eta = 0.1$, $\tilde{\mu} = 1$ and $\varepsilon' = 1$. In all the plots, we represented %O^{A} for the case in which only OCT4 is overexpressed (yellow curve). Definitions of the parameters $\bar{u}_O$, $\bar{u}_J$, ε_d, ε_e, $\tilde{\mu}'$, $\bar{p}_O$, $\bar{p}_J$, and $\bar{p}_T$ is given in the "Model of the epigenetic OCT4 gene regulatory network" subsection. For all simulations, we implemented the reactions listed in Figure 1 with the SSA [5], we considered a time span of 21 days ($\tau = 201.6$), and $D_{tot} = 50$ (see Supplementary Note 1).

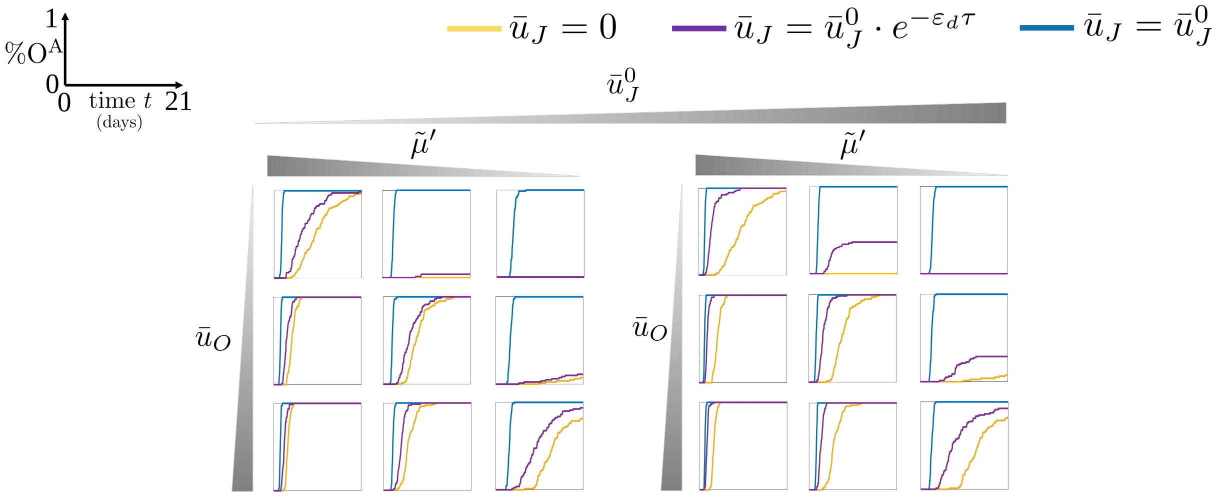

Supplementary Figure 10: **The effectiveness of transient overexpression of JMJD2 becomes more comparable to constant overexpression when the initial level of overexpression is higher.** $\%\mathrm{O}^{\mathrm{A}}$, that is, the normalized amount of $N = 100$ time trajectories which reach $n_O^A \geq 40$, starting from $n_O^A = 0$, for different values of $\bar{u}_T^0$. In all plots, on the x axis we have the time (days). The parameter values used for these simulations can be found in Supplementary table 6. In particular, we consider two values of $\bar{u}_J^0$ ($\bar{u}_J^0 = 160, 480$), three values of $\tilde{\mu}'$ ($\tilde{\mu}' = 1, 0.5, 0.2$), three values of $\bar{u}_O$ ($\bar{u}_O = 160, 320, 480$), and we set $\varepsilon = 0.3$, in which $\varepsilon = \varepsilon_d = \varepsilon_e$, $\bar{p} = 2.5$, in which $\bar{p} = \bar{p}_O = \bar{p}_T = \bar{p}_J$, $\eta = 0.1$, $\tilde{\mu} = 1$ and $\varepsilon' = 1$. In all the plots, we represented $\%\mathrm{O}^{\mathrm{A}}$ for the case in which only OCT4 is overexpressed (yellow curve). For all simulations, we implemented the reactions listed in Figure 1 with the SSA [5], we considered a time span of 21 days ($\tau = 201.6$), and $\mathrm{D}_{\mathrm{tot}} = 50$ (see Supplementary Note 1).

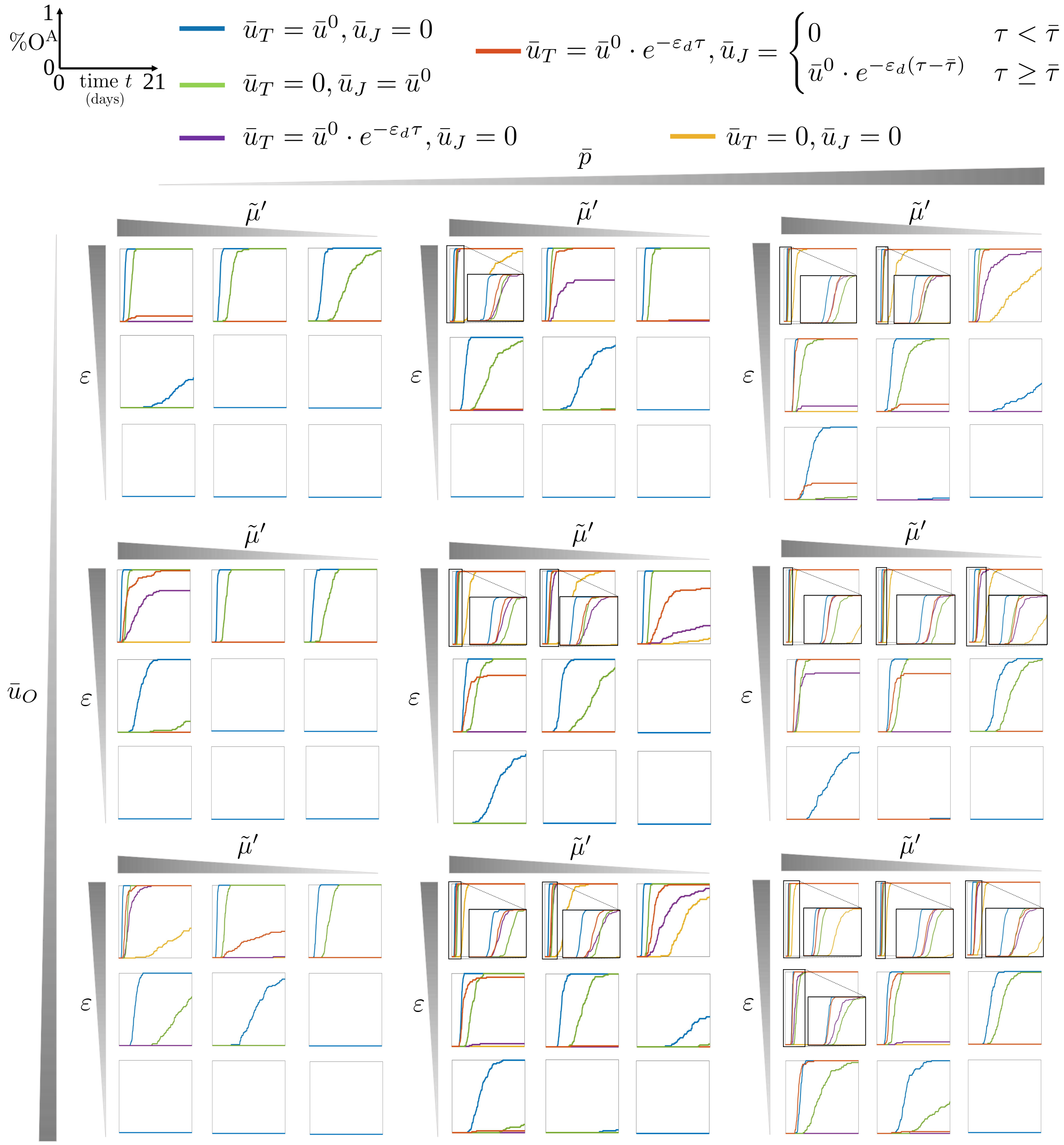

Supplementary Figure 11: **Comparison of the effect of sequential, transient overexpression of both epigenetic modifiers TET1 and JMJD2 and constant overexpression of a single epigenetic modifier on efficiency and latency variability of reprogramming through overexpression of OCT4 for different values of $\bar{u}_O$, ε_d, ε_e, $\tilde{\mu}'$, and $\bar{p}$.** $\%\mathrm{O}^\mathrm{A}$, that is, the normalized amount of $N = 100$ time trajectories which reach $n_O^A \geq 40$, starting from $n_O^A = 0$, for different parameter values. In all plots, on the x axis we have the time (days). The parameter values used for these simulations can be found in Supplementary table 5. In particular, we consider three values of $\tilde{\mu}'$ ($\tilde{\mu}' = 1, 0.5, 0.2$), three values of ε ($\varepsilon = 0.3, 0.1, 0.06$), in which $\varepsilon = \varepsilon_d = \varepsilon_e$, three values of $\bar{p}$ ($\bar{p} = 1, 2.5, 5$), in which $\bar{p} = \bar{p}_O = \bar{p}_T = \bar{p}_J$, three values of $\bar{u}_O$ ($\bar{u}_O = 160, 320, 480$), and we set $\bar{u}^0 = 160$, $\eta = 0.1$, $\tilde{\mu} = 1$ and $\varepsilon' = 1$. In all the plots, we represented $\%\mathrm{O}^\mathrm{A}$ for the case in which only OCT4 is overexpressed (yellow curve). For all simulations, we implemented the reactions listed in Figure 1 with the SSA [5], we considered a time span of 21 days ($\tau = 201.6$), and $\mathrm{D_{tot}} = 50$ (see Supplementary Note 1). Finally, for the sequential, transient overexpression case (red line), we introduced the first overepxression (TET1) at $t = 0$ ($\bar{\tau} = 0$) and the second overexpression (JMJD2) after 1 day ($\bar{\tau} = 12$).

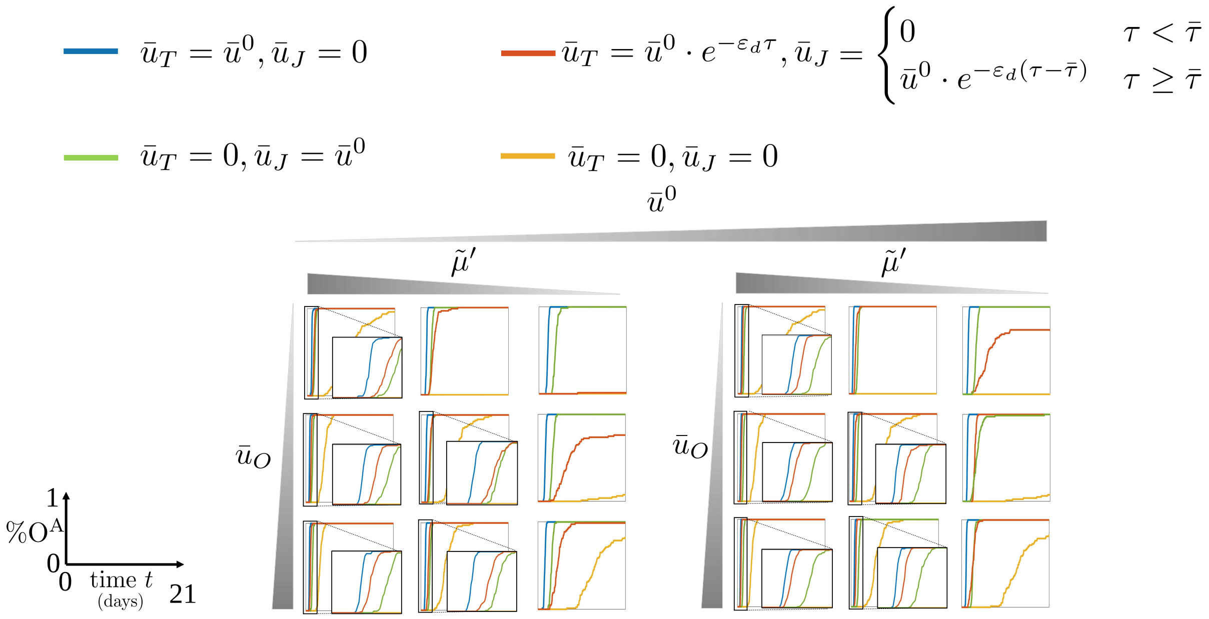

Supplementary Figure 12: **The effectiveness of sequential, transient overexpression of TET1 and JMJD2 increases when the initial level of overexpression is higher.** $\%\mathrm{O}^{\mathrm{A}}$, that is, the normalized amount of $N = 100$ time trajectories which reach $n_O^A \geq 40$, starting from $n_O^A = 0$, for different values of $\bar{u}^0$. In all plots, on the x axis we have the time (days). The parameter values used for these simulations can be found in Supplementary table 6. In particular, we consider two values of $\bar{u}^0$ ($\bar{u}_J^0 = 160, 320$), three values of $\tilde{\mu}'$ ($\tilde{\mu}' = 1, 0.5, 0.2$), three values of $\bar{u}_O$ ($\bar{u}_O = 160, 320, 480$), and we set $\varepsilon = 0.3$, in which $\varepsilon = \varepsilon_d = \varepsilon_e$, $\bar{p} = 2.5$, in which $\bar{p} = \bar{p}_O = \bar{p}_T = \bar{p}_J$, $\eta = 0.1$, $\tilde{\mu} = 1$ and $\varepsilon' = 1$. In all the plots, we represented $\%\mathrm{O}^{\mathrm{A}}$ for the case in which only OCT4 is overexpressed (yellow curve). For all simulations, we implemented the reactions listed in Figure 1 with the SSA [5], we considered a time span of 21 days ($\tau = 201.6$), and $\mathrm{D_{tot}} = 50$ (see Supplementary Note 1). Finally, for the sequential, transient overexpression case (red line), we introduced the first overepxression (TET1) at $t = 0$ ($\bar{\tau} = 0$) and the second overexpression (JMJD2) after 1 day ($\bar{\tau} = 12$).

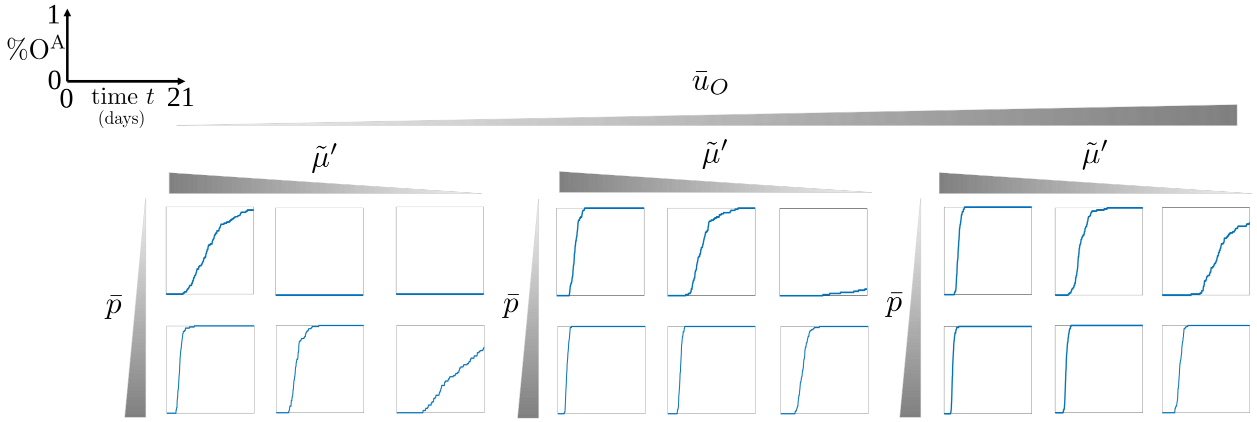

Supplementary Figure 13: **How the level of OCT4 overexpression ($\bar{u}_O$) affects the reprogramming process for different values of $\tilde{\mu}'$ and $\bar{p}$.** $\%\mathrm{O}^{\mathrm{A}}$, that is, the normalized amount of $N = 100$ time trajectories which reach $n_O^A \geq 40$, starting from $n_O^A = 0$, for different values of $\bar{u}_O$. In all plots, on the x axis we have the time (days). The parameter values used for these simulations can be found in Supplementary table 7. In particular, we consider three values of $\bar{u}_O$ ($\bar{u}_O = 160, 320, 480$), three values of $\tilde{\mu}'$ ($\tilde{\mu}' = 1, 0.5, 0.2$), two values of $\bar{p}$ ($\bar{p} = 2.5, 5$), in which $\bar{p} = \bar{p}_O = \bar{p}_T = \bar{p}_J$, and we set $\varepsilon = 0.3$, in which $\varepsilon = \varepsilon_d = \varepsilon_e$, $\eta = 0.1$, $\tilde{\mu} = 1$ and $\varepsilon' = 1$. For all simulations, we implemented the reactions listed in Figure 1 with the SSA [5], we considered a time span of 21 days ($\tau = 201.6$), and $\mathrm{D_{tot}} = 50$ (see Supplementary Note 1).

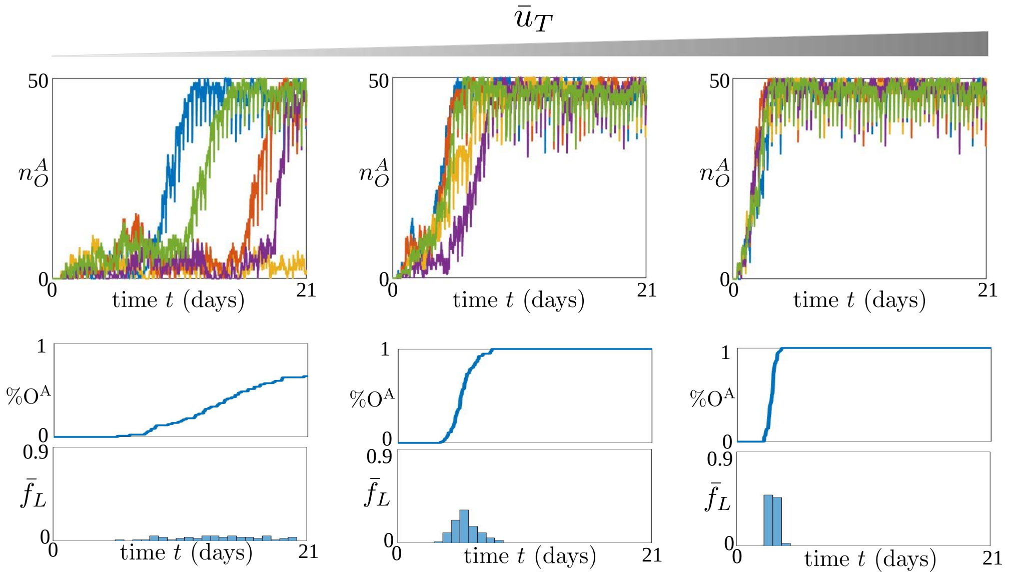

Supplementary Figure 14: **Effect of TET1 overexpression ($\bar{u}_T$) on the reprogramming process through overexpression of OCT4 by simulating the model including the binomial partitioning of molecules at the end of the cell cycle (Supplementary Note 4).** Top plots: time trajectories of n_O^A (total amount of nucleosomes modified with activating chromatin modifications for the OCT4 gene) starting from the OCT4 fully repressed state ($n_O^A = 0$) for different values of $\bar{u}_T$. Bottom plots: %O^{A}, that is, the normalized amount of $N = 100$ time trajectories which reach $n_O^A \geq 40$, starting from $n_O^A = 0$, and $\bar{f}_L$, that is, the normalized frequency of a specific latency value across all the $N = 100$ simulations. In all plots, on the x axis we have the time (days). The parameter values used for these simulations can be found in Supplementary table 8. In particular, we consider three values of $\bar{u}_T$ (i.e., $\bar{u}_T = 0, 50, 160$), and we set $\bar{u}_O = 320$, $\bar{u}_J = 0$, $\tilde{\mu}' = 0.5$, $\varepsilon_d = 0.2$, $\varepsilon_e = 0.2$, $\bar{p}_O = \bar{p}_T = \bar{p}_J = 5$, $\tilde{\mu} = 1$ and $\varepsilon' = 1$. For all simulations, we implemented the reaction systems described in Supplementary Note 4 with the ATG alghoritm [4] and we considered a time span of 21 days ($\tau = 201.6$) and $\mathrm{D_{tot}} = 50$ (see Supplementary Note 1).

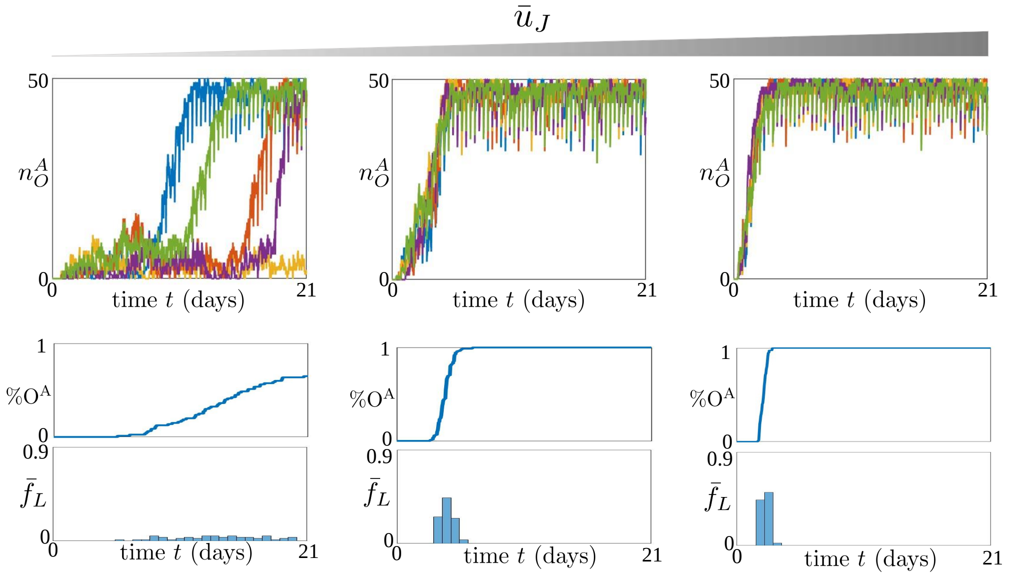

Supplementary Figure 15: **Effect of JMJD2 overexpression ($\bar{u}_J$) on the reprogramming process through overexpression of OCT4 by simulating the model including the binomial partitioning of molecules at the end of cell cycle (Supplementary Note 4).** Top plots: time trajectories of n_O^A (total amount of nucleosomes modified with activating chromatin modifications for the OCT4 gene) starting from the OCT4 fully repressed state ($n_O^A = 0$) for different values of $\bar{u}_J$. Bottom plots: $\%\mathrm{O}^\mathrm{A}$, that is, the normalized amount of $N = 100$ time trajectories which reach $n_O^A \geq 40$, starting from $n_O^A = 0$, and $\bar{f}_L$, that is, the normalized frequency of a specific latency value across all the $N = 100$ simulations. In all plots, on the x axis we have the time (days). The parameter values used for these simulations can be found in Supplementary table 8. In particular, we consider three values of $\bar{u}_T$ (i.e., $\bar{u}_J = 0, 50, 160$), and we set $\bar{u}_O = 320$, $\bar{u}_T = 0$, $\tilde{\mu}' = 0.5$, $\varepsilon_d = 0.2$, $\varepsilon_e = 0.2$, $\bar{p}_O = \bar{p}_T = \bar{p}_J = 5$, $\tilde{\mu} = 1$ and $\varepsilon' = 1$. For all simulations, we implemented the reaction systems described in Supplementary Note 4 with the ATG alghoritm [4] and we considered a time span of 21 days ($\tau = 201.6$) and $\mathrm{D}_{\mathrm{tot}} = 50$ (see Supplementary Note 1).

6 Parameter values and motivation of parameter choices used to generate the plots in the main paper and SI

In our model, $\mathrm{D_{tot}}$ represents the total number of nucleosomes within a gene of interest. Assuming about one nucleosome per 200 bp [1] and assuming that an average gene spans between 10,000 and 20,000 bp [2], then the value of $\mathrm{D_{tot}}$ can be considered, on average, between 50 and 100. In our computational study, we set $\mathrm{D_{tot}} = 50$.

In our model, we describe the effect of dilution due to cell growth and division as an effective decay reaction with first-order kinetics, which is one of the standard models used [3]. The assumption supporting this model is that cells grow exponentially in size [2], with growth rate constant $\delta > 0$. This means that species' concentration decays exponentially through a first-order reaction, with half-life that can be written as $t_{hl} = ln(2)/\delta$. Furthermore, if we also assume that, on average, the species concentration is evenly divided at cell division, then the half-life is equal to the cell cycle length T (i.e., $t_{hl} = T$), where T is the cell cycle length. The decay rate constant can then be written as $\delta = ln(2)/T$. Given that the mammalian cell cycle length is 16 - 24 hr [2, 6], we consider $\delta = 0.04$ (corresponding to $T = 17.33$ h) as an intermediate value for δ.

Furthermore, we set $\eta = 0.1$, and then $\delta' = 0.1\delta$, based on experimental data showing the high efficiency of the DNA methylation maintenance process through DNMT1 [7].

In [8], Hanna et al. show that the latency of silenced gene reactivation exhibits significant variability. Based on the original study conducted to characterize the chromatin modification circuit used here [9], it is observed that the reactivation process exhibits high latency variability within a parameter regime where ε_d is small. We then set $k_M^A/\Omega = 0.008\ \mathrm{h}^{-1}$, so that, in the range of δ values considered, $\varepsilon_d = (\delta)/(\frac{k_M^A}{\Omega}\mathrm{D_{tot}})$ spans between 0.06 and 0.3.

Concerning the rate of the recruited erasure process k_E^A/Ω, it is well known that the rates of enzymatic reactions characterized by highly specific enzyme-substrate binding tend to be higher than those of enzymatic reactions involving non-specific enzyme-substrate binding and removal through dilution resulting from cell growth [10]. Furthermore, previous studies show that varying the parameter ε' does not significantly affect the trends with which the other parameters affect the stochastic behavior of the chromatin modification circuit considered in this paper, unless ε' is much smaller than ε_d and ε_e [9]. Thus, we set k_E^A/Ω so that $\varepsilon' = (k_E^A/\Omega)/(k_M^A/\Omega) = 1$, that is, ε' is always larger than the values of ε_e and ε_d considered in our simulations.

Finally, recent experimental data show that the DNA methylation erasure process is significantly slower compared to histone modification erasure [11]. Thus, we set values for the erasure rates of DNA methylation always equal or lower compared to the erasure rates of repressive histone modifications, that is, setting $\tilde{k}_T'^*$ and $\tilde{k}_2^R$ such that $\tilde{\mu}'/\tilde{\mu} = \tilde{k}_T'^*/\tilde{k}_2^R \leq 1$.

Regarding the other parameters, multiple parameter regimes have been considered in order to better understand how different parameters affect the stochastic properties of the system.

**Param.**	**Value (h^{-1})** Figure 3	**Value (h^{-1})** Figure 4	**Value (h^{-1})** Figure 5a	**Value (h^{-1})** Figure 5(b)
k_{W0}^A	0.04	0.04	0.04	0.04
k_{W0}^1	0.04	0.04	0.04	0.04
k_{W0}^2	0.04	0.04	0.04	0.04
$\frac{\tilde{k}_W^A}{\Omega}$	4	4	4	4
$\bar{k}_E^A$	0.144	0.12	0.08	0.04
δ	0.144	0.12, 0.04, 0.024	0.08	0.04
$\frac{k_M^A}{\Omega}$	0.008	0.008	0.008	0.008
$\frac{k_E^A}{\Omega}$	0.008	0.008	0.008	0.008
$\frac{\hat{k}_T^{'}}{\Omega}$	2.9×10^{-3}, 4.32×10^{-4}, 2.02×10^{-4}	1.9×10^{-3}	8×10^{-4}	0.0024,4×10^{-4},1.6×10^{-4}
$\delta^{'}$	0.0144	0.012,0.004,0.0024	0.008	0.004
$\frac{k_M^{'}}{\Omega}$	0.0016	0.0016	0.0016	0.0016
$\frac{\tilde{k}_T^{'*}}{\Omega^2}$	1.6×10^{-4},2.4×10^{-5},1.12×10^{-5}	1.6×10^{-4}	8×10^{-5}	4.8×10^{-4},8×10^{-5},3.2×10^{-5}
$\frac{\tilde{k}_E^R}{\Omega}$	0.0029	0.0024	0.0016	0.0008
$\frac{k_M}{\Omega}$	0.008	0.008	0.008	0.008
$\frac{\bar{k}_M}{\Omega}$	0.0016	0.0016	0.0016	0.0016
$\frac{\tilde{k}_E^R}{\Omega^2}$	1.6×10^{-4}	1.6×10^{-4}	1.6×10^{-4}	1.6×10^{-4}
$\frac{a_e}{\Omega}$	0.008	0.008	0.008	0.008
d_e	0.8	0.8	0.8	0.8
$\frac{a_t^d}{\Omega}$	0.008	0.008	0.008	0.008
d_t^d	0.8	0.8	0.8	0.8
$K_A\Omega$	100	100	100	100
α_O	0.64	1	1	1
α_T	0.64	1	1	1
α_J	0.64	1	1	1
γ_O	0.2	0.2	0.2	0.2
γ_T	0.2	0.2	0.2	0.2
γ_J	0.2	0.2	0.2	0.2
$u_O\Omega$	0	64	64	64
$u_T\Omega$	0	0	0,10,32	0,10,32
$u_J\Omega$	0	0	0	0

Supplementary table 1: **Parameter values associated with the plots in Figures 3 - 5.**

**Param.**	**Value (h^{-1})** Figure 6a	**Value (h^{-1})** Figure 6(b)	**Value (h^{-1})** Supplementary Figure 1	**Value (h^{-1})** Supplementary Figure 2
k_{W0}^{A}	0.04	0.04	0.04	0.04
k_{W0}^{1}	0.04	0.04	0.04	0.04
k_{W0}^{2}	0.04	0.04	0.04	0.04
$\frac{\tilde{k}_W^A}{\Omega}$	4	4	4	4
$\bar{k}_E^A$	0.08	0.064	0.144	0.08
δ	0.08	0.064	0.144	0.08
$\frac{k_M^A}{\Omega}$	0.008	0.008	0.008	0.008
$\frac{k_E^A}{\Omega}$	0.008	0.008	0.008	0.008
$\frac{\hat{k}_T^{'}}{\Omega}$	8×10^{-4}	0.0013,6.4×10^{-4},1.28×10^{-4}	2.9×10^{-3}	8×10^{-4}
$\delta^{'}$	0.008	0.0064	0.0144	0.008
$\frac{k_M^{'}}{\Omega}$	0.0016	0.08,0.04,0.008,0.0013	0.0016	0.0016
$\frac{\tilde{k}_T^{'*}}{\Omega^2}$	8×10^{-5}	1.6×10^{-4},8×10^{-5},1.6×10^{-5}	1.6×10^{-4}	8×10^{-5}
$\frac{\tilde{k}_E^R}{\Omega}$	0.0016	0.0013	2.9×10^{-3},4.32×10^{-4},2.02×10^{-4}	0.0016
$\frac{k_M}{\Omega}$	0.008	0.008	0.008	0.008
$\frac{\bar{k}_M}{\Omega}$	0.0016	0.0016	0.0016	0.0016
$\frac{\tilde{k}_E^R}{\Omega^2}$	1.6×10^{-4}	1.6×10^{-4}	1.6×10^{-4}, 2.4×10^{-5},1.12×10^{-5}	1.6×10^{-4}
$\frac{a_e}{\Omega}$	0.008	0.008	0.008	0.008
d_e	0.8	0.8	0.8	0.8
$\frac{a_t^d}{\Omega}$	0.008	0.008	0.008	0.008
d_t^d	0.8	0.8	0.8	0.8
$K_A\Omega$	100	100	100	100
α_O	1	1	1	1
α_T	1	1	1	1
α_J	1	1	1	1
γ_O	0.2	0.2	0.2	0.2
γ_T	0.2	0.2	0.2	0.2
γ_J	0.2	0.2	0.2	0.2
$u_O\Omega$	64	32	0	32,44,64
$u_T\Omega$	0	0,64	0	0
$u_J\Omega$	0,10,32	0,64	0	0

Supplementary table 2: **Parameter values associated with the plots in Figure 6 and Supplementary Figure 1 - Supplementary Figure 2.**

**Param.**	**Value (h^{-1})** Supplementary Figure 3	**Value (h^{-1})** Supplementary Figure 4	**Value (h^{-1})** Supplementary Figure 5
k_{W0}^A	0.04	0.04	0.04
k_{W0}^1	0.04	0.04	0.04
k_{W0}^2	0.04	0.04	0.04
$\frac{\tilde{k}_W^A}{\Omega}$	4	4	4
$\bar{k}_E^A$	0.12,0.04,0.024	0.12,0.04,0.024	0.12,0.04,0.024
δ	0.12,0.04,0.024	0.12,0.04,0.024	0.12,0.04,0.024
$\frac{k_M^A}{\Omega}$	0.008	0.008	0.008
$\frac{k_E^A}{\Omega}$	0.008	0.008	0.008
$\frac{\hat{k}_T^{'}}{\Omega}$	$0.0024,8\times10^{-4},4.8\times10^{-4}$	$0.0024,8\times10^{-4},4.8\times10^{-4}$	$0.0024,8\times10^{-4},4.8\times10^{-4}$ (top line of each subpanel)
	$0.0012,4\times10^{-4},2.4\times10^{-4}$	$0.0012,4\times10^{-4},2.4\times10^{-4}$	$0.0012,4\times10^{-4},2.4\times10^{-4}$ (intermediate line of each subpanel)
	$4.8\times10^{-4},1.6\times10^{-4},9.6\times10^{-5}$	$4.8\times10^{-4},1.6\times10^{-4},9.6\times10^{-5}$	$4.8\times10^{-4},1.6\times10^{-4},9.6\times10^{-5}$ (bottom line of each subpanel)
$\delta^{'}$	0.012,0.004,0.0024	0.012,0.004,0.0024	0.012,0.004,0.0024
$\frac{k_M^{'}}{\Omega}$	0.0016	0.0016	0.0016
$\frac{\tilde{k}_T^{'*}}{\Omega^2}$	$1.6\times10^{-4},8\times10^{-5},3.2\times10^{-5}$	$1.6\times10^{-4},8\times10^{-5},3.2\times10^{-5}$	$1.6\times10^{-4},8\times10^{-5},3.2\times10^{-5}$
$\frac{\hat{k}_E^R}{\Omega}$	$0.0024,8\times10^{-4},4.8\times10^{-4}$	$0.0024,8\times10^{-4},4.8\times10^{-4}$	$0.0024,8\times10^{-4},4.8\times10^{-4}$
$\frac{k_M}{\Omega}$	0.008	0.008	0.008
$\frac{\bar{k}_M}{\Omega}$	0.0016	0.0016	0.0016
$\frac{\tilde{k}_E^R}{\Omega^2}$	1.6×10^{-4}	1.6×10^{-4}	1.6×10^{-4}
$\frac{a_e}{\Omega}$	0.008	0.008	0.008
d_e	0.8	0.8	0.8
$\frac{a_t^d}{\Omega}$	0.008	0.008	0.008
d_t^d	0.8	0.8	0.8
$K_A\Omega$	100	100	100
α_O	1,0.5,0.2	1,0.5,0.2	1,0.5,0.2
α_T	1,0.5,0.2	1,0.5,0.2	1,0.5,0.2
α_J	1,0.5,0.2	1,0.5,0.2	1,0.5,0.2
γ_O	0.2	0.2	0.2
γ_T	0.2	0.2	0.2
γ_J	0.2	0.2	0.2
$u_O\Omega$	32,64,96	32,64,96	32,64,96
$u_T\Omega$	0	0,10,32	0
$u_J\Omega$	0	0	0,10,32

Supplementary table 3: **Parameter values associated with the plots in Supplementary Figure 3, Supplementary Figure 4, and Supplementary Figure 5.**

**Param.**	**Value (h^{-1})**<br>Supplementary Figure 6
k_{W0}^{A}	0.04
k_{W0}^{1}	0.04
k_{W0}^{2}	0.04
$\frac{\tilde{k}_W^A}{\Omega}$	4
$\bar{k}_E^A$	0.12,0.096,0.064
δ	0.12,0.096,0.064
$\frac{k_M^A}{\Omega}$	0.008
$\frac{k_E^A}{\Omega}$	0.008
$\frac{\hat{k}_T^{'}}{\Omega}$	0.0024,0.0012,2.4×10^{-4} (left-hand side panels)<br>0.0019,9.6×10^{-4},1.92×10^{-4} (central panels)<br>0.0013,6.4×10^{-4},1.28×10^{-4} (right-hand side panels)
δ'	0.012,0.0096,0.0064
$\frac{k_M^{'}}{\Omega}$	0.04,0.008,0.0016
$\frac{\tilde{k}_T^{'*}}{\Omega^2}$	1.6×10^{-4},8×10^{-5},1.6×10^{-5}
$\frac{\hat{k}_E^R}{\Omega}$	0.0024,0.0019,0.0013
$\frac{k_M}{\Omega}$	0.008
$\frac{\bar{k}_M}{\Omega}$	0.0016
$\frac{\tilde{k}_E^R}{\Omega^2}$	1.6×10^{-4}
$\frac{a_e}{\Omega}$	0.008
d_e	0.8
$\frac{a_t^d}{\Omega}$	0.008
d_t^d	0.8
$K_A\Omega$	100
α_O	1
α_T	1
α_J	1
γ_O	0.2
γ_T	0.2
γ_J	0.2
$u_O\Omega$	32
$u_T\Omega$	0,20,32,64
$u_J\Omega$	0,20,32,64

Supplementary table 4: **Parameter values associated with the plots in Supplementary Figure 6.**

**Param.**	**Value (h^{-1})** Supplementary Figure 7	**Value (h^{-1})** Supplementary Figure 9	**Value (h^{-1})** Supplementary Figure 11
k_{W0}^A	0.04	0.04	0.04
k_{W0}^1	0.04	0.04	0.04
k_{W0}^2	0.04	0.04	0.04
$\frac{\tilde{k}_W^A}{\Omega}$	4	4	4
$\bar{k}_E^A$	0.12,0.04,0.024	0.12,0.04,0.024	0.12,0.04,0.024
δ	0.12,0.04,0.024	0.12,0.04,0.024	0.12,0.04,0.024
$\frac{k_M^A}{\Omega}$	0.008	0.008	0.008
$\frac{k_E^A}{\Omega}$	0.008	0.008	0.008
$\frac{\hat{k}_T'}{\Omega}$	$0.0024, 8\times10^{-4}, 4.8\times10^{-4}$	$0.0024, 8\times10^{-4}, 4.8\times10^{-4}$	$0.0024, 8\times10^{-4}, 4.8\times10^{-4}$ (top line of each subpanel)
	$0.0012, 4\times10^{-4}, 2.4\times10^{-4}$	$0.0012, 4\times10^{-4}, 2.4\times10^{-4}$	$0.0012, 4\times10^{-4}, 2.4\times10^{-4}$ (intermediate line of each subpanel)
	$4.8\times10^{-4}, 1.6\times10^{-4}, 9.6\times10^{-5}$	$4.8\times10^{-4}, 1.6\times10^{-4}, 9.6\times10^{-5}$	$4.8\times10^{-4}, 1.6\times10^{-4}, 9.6\times10^{-5}$ (bottom line of each subpanel)
δ'	0.012,0.004,0.0024	0.012,0.004,0.0024	0.012,0.004,0.0024
$\frac{k_M'}{\Omega}$	0.0016	0.0016	0.0016
$\frac{\tilde{k}_T'^*}{\Omega^2}$	$1.6\times10^{-4}, 8\times10^{-5}, 3.2\times10^{-5}$	$1.6\times10^{-4}, 8\times10^{-5}, 3.2\times10^{-5}$	$1.6\times10^{-4}, 8\times10^{-5}, 3.2\times10^{-5}$
$\frac{\hat{k}_E^R}{\Omega}$	$0.0024, 8\times10^{-4}, 4.8\times10^{-4}$	$0.0024, 8\times10^{-4}, 4.8\times10^{-4}$	$0.0024, 8\times10^{-4}, 4.8\times10^{-4}$
$\frac{k_M}{\Omega}$	0.008	0.008	0.008
$\frac{\bar{k}_M}{\Omega}$	0.0016	0.0016	0.0016
$\frac{\tilde{k}_E^R}{\Omega^2}$	1.6×10^{-4}	1.6×10^{-4}	1.6×10^{-4}
$\frac{a_e}{\Omega}$	0.008	0.008	0.008
d_e	0.8	0.8	0.8
$\frac{a_t^d}{\Omega}$	0.008	0.008	0.008
d_t^d	0.8	0.8	0.8
$K_A\Omega$	100	100	100
α_O	1,0.5,0.2	1,0.5,0.2	1,0.5,0.2
α_T	1,0.5,0.2	1,0.5,0.2	1,0.5,0.2
α_J	1,0.5,0.2	1,0.5,0.2	1,0.5,0.2
γ_O	0.2	0.2	0.2
γ_T	0.2	0.2	0.2
γ_J	0.2	0.2	0.2
$u_O\Omega$	32,64,96	32,64,96	32,64,96
$u_T\Omega$	$0, 32, 32\cdot e^{-\delta t}$	0	$0, 32, 32\cdot e^{-\delta t}$
$u_J\Omega$	0	$0, 32, 32\cdot e^{-\delta t}$	$0, 32, 32\cdot e^{-\delta t}$

Supplementary table 5: **Parameter values associated with the plots in Supplementary Figure 7, Supplementary Figure 9, and Supplementary Figure 11.**

**Param.**	**Value (h^{-1})** Supplementary Figure 8	**Value (h^{-1})** Supplementary Figure 10	**Value (h^{-1})** Supplementary Figure 12
k_{W0}^A	0.04	0.04	0.04
k_{W0}^1	0.04	0.04	0.04
k_{W0}^2	0.04	0.04	0.04
$\frac{\tilde{k}_W^A}{\Omega}$	4	4	4
$\bar{k}_E^A$	0.12,0.04,0.024	0.12,0.04,0.024	0.12,0.04,0.024
δ	0.12,0.04,0.024	0.12,0.04,0.024	0.12,0.04,0.024
$\frac{k_M^A}{\Omega}$	0.008	0.008	0.008
$\frac{k_E^A}{\Omega}$	0.008	0.008	0.008
$\frac{\hat{k}_T'}{\Omega}$	0.0024,8×10^{-4},4.8×10^{-4}	0.0024,8×10^{-4},4.8×10^{-4}	0.0024,8×10^{-4},4.8×10^{-4} (top line of each subpanel)
	0.0012,4×10^{-4},2.4×10^{-4}	0.0012,4×10^{-4},2.4×10^{-4}	0.0012,4×10^{-4},2.4×10^{-4} (intermediate line of each subpanel)
	4.8×10^{-4},1.6×10^{-4},9.6×10^{-5}	4.8×10^{-4},1.6×10^{-4},9.6×10^{-5}	4.8×10^{-4},1.6×10^{-4},9.6×10^{-5} (bottom line of each subpanel)
δ'	0.012,0.004,0.0024	0.012,0.004,0.0024	0.012,0.004,0.0024
$\frac{k_M'}{\Omega}$	0.0016	0.0016	0.0016
$\frac{\tilde{k}_T'^{*}}{\Omega^2}$	1.6×10^{-4},8×10^{-5},3.2×10^{-5}	1.6×10^{-4},8×10^{-5},3.2×10^{-5}	1.6×10^{-4},8×10^{-5},3.2×10^{-5}
$\frac{\hat{k}_E^R}{\Omega}$	0.0024,8×10^{-4},4.8×10^{-4}	0.0024,8×10^{-4},4.8×10^{-4}	0.0024,8×10^{-4},4.8×10^{-4}
$\frac{k_M}{\Omega}$	0.008	0.008	0.008
$\frac{\bar{k}_M}{\Omega}$	0.0016	0.0016	0.0016
$\frac{\tilde{k}_E^R}{\Omega^2}$	1.6×10^{-4}	1.6×10^{-4}	1.6×10^{-4}
$\frac{a_e}{\Omega}$	0.008	0.008	0.008
d_e	0.8	0.8	0.8
$\frac{a_t^d}{\Omega}$	0.008	0.008	0.008
d_t^d	0.8	0.8	0.8
$K_A\Omega$	100	100	100
α_O	1,0.5,0.2	1,0.5,0.2	1,0.5,0.2
α_T	1,0.5,0.2	1,0.5,0.2	1,0.5,0.2
α_J	1,0.5,0.2	1,0.5,0.2	1,0.5,0.2
γ_O	0.2	0.2	0.2
γ_T	0.2	0.2	0.2
γ_J	0.2	0.2	0.2
$u_O\Omega$	32,64,96	32,64,96	32,64,96
$u_T\Omega$	0,32,32$\cdot e^{-\delta t}$	0	0,32,32$\cdot e^{-\delta t}$ (left-hand side plots)
	0,64,64$\cdot e^{-\delta t}$	0	0,64,64$\cdot e^{-\delta t}$ (right-hand side plots)
$u_J\Omega$	0	0,32,32$\cdot e^{-\delta t}$	0,32,32$\cdot e^{-\delta t}$ (left-hand side plots)
	0	0,96,96$\cdot e^{-\delta t}$	0,64,64$\cdot e^{-\delta t}$ (right-hand side plots)

Supplementary table 6: **Parameter values associated with the plots in Supplementary Figure 8, Supplementary Figure 10, and Supplementary Figure 12.**

**Param.**	**Value ($\mathbf{h}^{-1}$)**<br>Supplementary Figure 13
k_{W0}^{A}	0.04
k_{W0}^{1}	0.04
k_{W0}^{2}	0.04
$\frac{\tilde{k}_W^A}{\Omega}$	4
$\bar{k}_E^A$	0.12
δ	0.12
$\frac{k_M^A}{\Omega}$	0.008
$\frac{k_E^A}{\Omega}$	0.008
$\frac{\hat{k}_T^{'}}{\Omega}$	$0.0024, 8\times10^{-4}, 4.8\times10^{-4}$
$\delta^{'}$	0.012
$\frac{k_M^{'}}{\Omega}$	0.0016
$\frac{\tilde{k}_T^{'*}}{\Omega^2}$	$1.6\times10^{-4}, 8\times10^{-5}, 3.2\times10^{-5}$
$\frac{\hat{k}_E^R}{\Omega}$	0.0024
$\frac{k_M}{\Omega}$	0.008
$\frac{\bar{k}_M}{\Omega}$	0.0016
$\frac{\tilde{k}_E^R}{\Omega^2}$	1.6×10^{-4}
$\frac{a_e}{\Omega}$	0.008
d_e	0.8
$\frac{a_t^d}{\Omega}$	0.008
d_t^d	0.8
$K_A\Omega$	100
α_O	1,0.5
α_T	1,0.5
α_J	1,0.5
γ_O	0.2
γ_T	0.2
γ_J	0.2
$u_O\Omega$	32,64,96
$u_T\Omega$	0
$u_J\Omega$	0

Supplementary table 7: **Parameter values associated with the plots in Supplementary Figure 13.**

**Param.**	**Value (h^{-1})** Supplementary Figure 14	**Value (h^{-1})** Supplementary Figure 15
k_{W0}^{A}	0.04	0.04
k_{W0}^{1}	0.04	0.04
k_{W0}^{2}	0.04	0.04
$\frac{\tilde{k}_W^A}{\Omega}$	4	4
$\bar{k}_E^A$	0.08	0.08
$\frac{k_M^A}{\Omega}$	0.008	0.008
$\frac{k_E^A}{\Omega}$	0.008	0.008
$\frac{\hat{k}_T^{'}}{\Omega}$	8×10^{-4}	8×10^{-4}
k_M^{DNMT1}	0.036	0.036
$\frac{k_M^{'}}{\Omega}$	0.0016	0.0016
$\frac{\tilde{k}_T^{'*}}{\Omega^2}$	8×10^{-5}	8×10^{-5}
$\frac{\hat{k}_E^R}{\Omega}$	0.0016	0.0016
$\frac{k_M}{\Omega}$	0.008	0.008
$\frac{\bar{k}_M}{\Omega}$	0.0016	0.0016
$\frac{\tilde{k}_E^R}{\Omega^2}$	1.6×10^{-4}	1.6×10^{-4}
$\frac{a_e}{\Omega}$	0.008	0.008
d_e	0.8	0.8
$\frac{a_t^d}{\Omega}$	0.008	0.008
d_t^d	0.8	0.8
$K_A\Omega$	100	100
α_O	1	1
α_T	1	1
α_J	1	1
γ_O	0.12	0.12
γ_T	0.12	0.12
γ_J	0.12	0.12
$u_O\Omega$	64	64
$u_T\Omega$	0,10,32	0
$u_J\Omega$	0	0,10,32

Supplementary table 8: **Parameter values associated with the plots in Supplementary Figure 14 and Supplementary Figure 15.**

References

1. Felsenfeld, G. & Groudine, M. Controlling the double helix. *Nature* **421** (2003).
2. Cooper, G. M. *The Cell: A Molecular Approach* (Sunderland (MA): Sinauer Associates, 2000).
3. Friedman, N., Cai, L. & Xie, X. S. Linking Stochastic Dynamics to Population Distribution: An Analytical Framework of Gene Expression. *Phys. Rev. Lett* **97** (2006).
4. Lu, T., Volfson, D., Tsimring, L. & Hasty, J. Cellular growth and division in the Gillespie algorithm. *Syst Biol (Stevenage)* **1,** 121–8 (2004).
5. Gillespie, D. T. Stochastic Simulation of Chemical Kinetics. *Annual Review of Physical Chemistry* **58,** 35–55 (2007).
6. J, H. *et al.* Human embryonic stem cells with biological and epigenetic characteristics similar to those of mouse ESCs. *Proc Natl Acad Sci* **107,** 9222–7 (2010).
7. Von Meyenn, F. *et al.* Impairment of DNA Methylation Maintenance Is the Main Cause of Global Demethylation in Naive Embryonic Stem Cells. *Mol. Cell* **62** (2016).
8. Hanna, J. *et al.* Direct cell reprogramming is a stochastic process amenable to acceleration. *Nature* **462** (2009).
9. Bruno, S., Williams, R. J. & Del Vecchio, D. Epigenetic cell memory: The gene's inner chromatin modification circuit. *PLOS Computational Biology* **18,** 1–27 (2022).
10. Bisswanger, H. *Enzyme Kinetics. Principles and Methods. 2nd Ed* (WILEY-VCH Verlag GmbH & Co. KGaA, Weinheim, 2008).

11. Bintu, L. *et al.* Dynamics of epigenetic regulation at the single-cell level. *Science* (2016).
